# Dysregulation of miRNA expression and excitation in MEF2C autism patient hiPSC-neurons and cerebral organoids

**DOI:** 10.1038/s41380-024-02761-9

**Published:** 2024-09-30

**Authors:** Dorit Trudler, Swagata Ghatak, Michael Bula, James Parker, Maria Talantova, Melissa Luevanos, Sergio Labra, Titas Grabauskas, Sarah Moore Noveral, Mayu Teranaka, Emily Schahrer, Nima Dolatabadi, Clare Bakker, Kevin Lopez, Abdullah Sultan, Parth Patel, Agnes Chan, Yongwook Choi, Riki Kawaguchi, Pawel Stankiewicz, Ivan Garcia-Bassets, Piotr Kozbial, Michael G. Rosenfeld, Nobuki Nakanishi, Daniel H. Geschwind, Shing Fai Chan, Wei Lin, Nicholas J. Schork, Rajesh Ambasudhan, Stuart A. Lipton

**Affiliations:** 1 Neurodegeneration New Medicines Center and Department of Molecular Medicine, The Scripps Research Institute, La Jolla, CA USA; 2 Neurodegenerative Disease Center, Scintillon Institute, San Diego, CA USA; 3 School of Biological Sciences, National Institute of Science Education and Research (NISER)-Bhubaneswar, an Off Campus Center of Homi Bhabha National Institute, Jatani, Odisha India; 4 Department of Neurosciences, University of California, San Diego, School of Medicine, La Jolla, CA USA; 5 Translational Genomics Research Institute, Phoenix, AZ USA; 6 Departments of Psychiatry and Neurology, David Geffen School of Medicine, University of California, Los Angeles, CA USA; 7 Department of Molecular and Human Genetics, Baylor College of Medicine, Houston, TX USA; 8 Howard Hughes Medical Institute, School and Department of Medicine, University of California, San Diego School of Medicine, La Jolla, CA USA; 9 Department of Neurology, Center for Autism Research and Treatment, Program in Neurobehavioral Genetics, Department of Human Genetics, Department of Psychiatry, Semel Institute, David Geffen School of Medicine, University of California, Los Angeles, CA USA; 10 Center for Neuroscience, Aging, and Stem Cell Research, Sanford Burnham Prebys Medical Discovery Institute, La Jolla, CA USA; 11 Department of Integrative Structural and Computational Biology, The Scripps Research Institute, La Jolla, CA USA; 12 Present Address: Department of Medicine, Indiana University-Purdue University, Indianapolis, IN USA

**Keywords:** Neuroscience, Autism spectrum disorders

## Abstract

MEF2C is a critical transcription factor in neurodevelopment, whose loss-of-function mutation in humans results in MEF2C haploinsufficiency syndrome (MHS), a severe form of autism spectrum disorder (ASD)/intellectual disability (ID). Despite prior animal studies of *MEF2C* heterozygosity to mimic MHS, MHS-specific mutations have not been investigated previously, particularly in a human context as hiPSCs afford. Here, for the first time, we use patient hiPSC-derived cerebrocortical neurons and cerebral organoids to characterize MHS deficits. Unexpectedly, we found that decreased neurogenesis was accompanied by activation of a micro-(mi)RNA-mediated gliogenesis pathway. We also demonstrate network-level hyperexcitability in MHS neurons, as evidenced by excessive synaptic and extrasynaptic activity contributing to excitatory/inhibitory (E/I) imbalance. Notably, the predominantly extrasynaptic (e)NMDA receptor antagonist, NitroSynapsin, corrects this aberrant electrical activity associated with abnormal phenotypes. During neurodevelopment, MEF2C regulates many ASD-associated gene networks, suggesting that treatment of MHS deficits may possibly help other forms of ASD as well.

## Introduction

Autism spectrum disorder (ASD) is a group of neurodevelopmental disabilities characterized by impaired social interaction and communication, accompanied by stereotyped behaviors and restricted interests [1]. It is estimated that 1 in 36 American children develop ASD [1, 2]. Despite extensive research that identified various genetic mutations (e.g., *MECP2*, *SHANK1-3*, *NRXN1-3*, etc.) and environmental factors, which are contributory to its etiology [3, 4], the molecular mechanisms underlying ASD remain largely unknown, hindering the development of robust diagnostics and effective therapies in this field.

Myocyte enhancer factor 2 (MEF2) comprises a family of transcription factors, which belong to the MADS (MCM1-agamous-deficiens-serum response factor) gene family [5–9], with four paralogs that play a role in development, cell differentiation, and organogenesis [10]. After discovering the MEF2C paralog and showing it is the first family member expressed during brain development [5, 6], we and others demonstrated its role in neurogenesis, synaptogenesis, and neuronal survival [11**–**14]. Subsequently, murine brain-specific knockout of *MEF2C* at early developmental stages was shown to display electrophysiological, histological, and behavioral deficits reminiscent of Rett syndrome (RTT), a severe form of ASD and intellectual disability (ID) [11, 15, 16]. In fact, the gene responsible for RTT, *MECP2*, is known to influence the expression level of MEF2C and vice-versa [17, 18].

Human genetic studies subsequently established an association between *MEF2C* and human ASD/ID. Morrow et al. identified various MEF2 target genes in their screen for autism genes in human pedigrees with shared ancestry [19]. Moreover, MEF2C activity transcriptionally regulates many other genes that have been linked to ASD [20, 21]. Recently, human studies from multiple laboratories pointed to defects in *MEF2C* itself in ASD, and *MEF2C* haploinsufficiency was identified as the cause for a severe form of ASD/ID in patients with little or no speech output, stereotypic movements, and learning/memory disorder, often accompanied by epilepsy [22**–**25]. These studies estimated that as many as 1.1% of patients with ASD or ID manifest a *MEF2C* abnormality as an autosomal dominant cause. Disorders associated with *MEF2C* haploinsufficiency have been collectively termed *MEF2C* haploinsufficiency syndrome (MHS) [25]. Importantly, recent comprehensive transcriptome analyses of autistic brains identified *MEF2C* as one of the frequently dysregulated genes in children with ASD even without *MEF2* mutations per se [20, 21, 26, 27]. For example, a histone-wide association study suggested that the MEF2C locus is dysregulated in ASD patients with other etiologies in addition to MHS [28]. Collectively, these data argue that finding a successful treatment for the MEF2C haploinsufficiency form of ASD may in fact benefit other forms of ASD as well.

Despite prior animal studies of *MEF2C* heterozygosity to mimic MHS [15, 29, 30], mechanisms whereby MEF2C affects neuronal development in the human context of ASD are still poorly understood. MEF2C is expressed in the brain in neurons and in microglia, but not in other cell types to any extent [5, 10, 11, 15, 29, 30]. Moreover, the effects of MHS-specific mutations have not been studied previously. Accordingly, here we used MHS patient-derived human induced pluripotent stem cells (hiPSCs) and CRISPR/Cas9 technology for isogenic controls to generate cerebrocortical neurons in 2-dimensional (2D) cultures and 3D cerebral organoids. These mutations manifest varying degrees of haploinsufficiency and are disease-specific. This approach allowed us to study human neuronal development and function in the presence of patient-relevant mutations in MEF2C-deficient compared to control cells, and thus to elucidate molecular and electrophysiological mechanisms underlying MHS pathophysiology.

## Materials and methods

### Materials: experimental model details

#### hiPSC reprogramming and maintenance

hiPSCs were generated from patient fibroblasts, by using an integration-free reprogramming method [31], which uses three episomal vectors that collectively encode OCT3/4, SOX2, KLF4, L-MYC, LIN28, and p53-shRNA. All lines except for control (Ctrl)3 (obtained from Coriell) were generated using the identical method of non-integrating episomal transfection and all lines were cultured and maintained under the same conditions. hiPSCs were characterized for pluripotency, karyotypic integrity and generation of all three germ layers. Karyotyping integrity was assessed for all patient lines and control lines by standard G-banding chromosome and DNA fingerprinting analysis at passage 15–20 after reprogramming by Cell Line Genetics (Madison, WI) or the commercial source from which the hiPSC line was obtained. hiPSCs were routinely cultured and maintained in our laboratory using a protocol described previously [32] with some modifications. Briefly, hiPSCs were plated on Matrigel coated plates (Corning, #354248) and cultured using mTeSR1 (STEMCELL Technologies, #05850) changed daily. The colonies were manually passaged weekly, using StemPro™ EZPassage™ Disposable Stem Cell Passaging Tool (Thermo-Fisher Scientific, #23181010).

#### Generation of MEF2C deletion by CRISPR/Cas9

Ctrl1 hiPSCs (Hs27, ATCC #CRL-1634; see Table S1) were used to produce the MEF2C deletion isogenic line at the Yale Stem Cell Center, which used CRISPR/Cas9 to generate an 11 bp deletion in MEF2C. Single clones were picked and further sequenced by Sanger sequencing. An isogenic line with an 11 bp deletion was further characterized by whole-exome gene sequencing. No off-target effects were detected in coding regions. The isogenic line also maintained its pluripotency.

#### Neuronal differentiation from hiPSCs

Differentiation of hiPSCs was performed using standard protocols for generating cerebrocortical neurons [32, 33]. Briefly, feeder-free hiPSCs cultured on Matrigel were induced to differentiate by exposure for 2 weeks to a cocktail of small molecules: 2 μM each of A83-01, dorsomorphin, and PNU74654 in DMEM/F12 medium supplemented with 20% Knock Out Serum Replacement (Invitrogen). Cells were then scraped manually to form floating neurospheres and maintained for 2 weeks in DMEM/F12 medium supplemented with N2 and B27 (Invitrogen) and 20 ng ml^−1^ of basic FGF (R&D Systems). Subsequently, neurospheres were seeded on polyornithine/laminin-coated dishes to form rosettes that were manually picked, expanded and frozen down as human neural progenitor cells (hNPCs). For terminal differentiation into neurons, hNPCs were plated onto polyornithine/laminin-coated glass coverslips or plates at a density of 1.5 × 10^6^ cells/cm^2^ in DMEM/F12 medium supplemented with N2 and B27. For the first 2 days, 0.1 µM of compound E (γ secretase inhibitor, CAS 209986-17-4, Calbiochem) was added. Starting on the third day, the medium was supplemented with GDNF (20 ng ml^−1^) and BDNF (20 ng ml^−1^).

For electrophysiological analysis and RNA-seq experiments, hNPCs were plated at a 1:1 ratio with neonatal mouse astrocytes onto polyornithine/laminin-coated glass coverslips in DMEM/F12 medium supplemented with B27, N2, GDNF (20 ng ml^−1^), and BDNF (20 ng ml^−1^) (Peprotech), and 0.5% FBS (Invitrogen). Prior to electrophysiology experiments, cells at week 3 of terminal differentiation were switched to BrainPhys medium (STEMCELL Technologies), and experiments were conducted after a total of 5–6 weeks of differentiation. Our protocol produced 8–15% inhibitory neurons, as monitored by immunocytochemistry with anti-GABA antibody [32].

#### hiPSC-derived cerebral organoid cultures

Cerebral organoids were generated with a protocol modified from the laboratory of Sergiu Pașca [34], and in our hands by scRNA-seq data and other criteria these organoids reliably expressed a population of GABAergic inhibitory neurons and their ventral progenitors in addition to a glutamatergic population of excitatory neurons and other cell types, including intermediate progenitors, astrocytes, and oligodendrocyte precursor cells (Fig. S9, Table S5). In brief, spheroids were generated from hiPSCs using SMAD inhibitors, dorsomorphin and SB-431542 for 5 days. From day 6, spheroids were transferred to Neurobasal medium supplemented with EGF and FGF-2 for 19 days. From day 25, the medium was supplemented with BDNF and NT3 until day 43, and from then on, the organoids were maintained in Neurobasal medium only. Cerebral organoids were maintained on an orbital shaker until use in experiments, as described below. Histology was performed after 2 to 3 months of differentiation. At 3–4 months of age, the cerebral organoids were used for multielectrode array (MEA) analysis. This 3–4 month interval was chosen for analysis here because exploratory data showed insufficient cell types were expressed in the isogenic control cells prior to the 3-month time point.

In this study, we used four patient-related MEF2C haploinsufficient (MHS) mutations and three control hiPSC lines, including a line isogenic/gene corrected to one of the patient-related lines. Due to the nature of growing the hiPSC cultures and organoids, with the extensive quality control (QC) involved in every batch with electrophysiological recordings, immunostaining, reporter gene assays, qPCR, and RNA-seq, not every control was available for every experiment. That said, we always ran multiple controls for each experiment, and the most critical control, the isogenic/gene-corrected control was always used for every experiment. Moreover, the controls all gave similar results, adding to our confidence that sufficient controls were used for each experimental paradigm.

#### Primary astrocytes

Primary astrocytes cultures were prepared as previously described [35]. Mice used for astrocyte cultures were housed and maintained in the animal facility at the Scintillon Institute or The Scripps Research Institute, and all experiments complied with protocols approved by the Institute Animal Care Committee (IACUC protocol, 17-0022-2). The brains from 1- to 2-day-old C57BL/6 mouse pups were rapidly harvested, and the meninges were removed. Brains were dissociated using mechanical means (pipettes and scissors) and enzymatic dissociation (0.25% trypsin for 30 min at 37 °C). The cell suspension was then filtered through a 70-µm cell strainer and cultured in DMEM:F12 medium supplemented with 10% fetal bovine serum, 100 µ/ml penicillin, and 0.1 mg/ml streptomycin. Two weeks after initial isolation, cells were dissociated with Accutase (STEMCELL Technologies, #07920), and the suspension was seeded onto glass coverslips coated with 20 µg/cm^2^ poly-l-ornithine (Sigma-Aldrich, #P4638), 2 µg/cm^2^ laminin (Trevigen, #3400-010-01), and 2 µg/cm^2^ fibronectin (Trevigen, #3420-001-01) at a density of 25,000 cells/cm^2^.

## Methods

### Immunoblots

Cells were lysed with cell lysis buffer (Cell Signaling Technologies, #9803) supplemented with protease inhibitor cocktail (Roche, #04693159001) and 10 µM SDS. Protein concentrations were determined using Pierce™ BCA Protein Assay Kit (Thermo-Fisher Scientific, #23225). Polyacrylamide gels (Novex, 8–12%) were used for protein separation prior to transfer onto nitrocellulose membrane, blockade with blocking buffer (Li-Cor) for 1 h, washing with TBST, and reaction overnight at 4 °C with mouse anti-MAP2 (1:2,000; Sigma-Aldrich, #M4403, RRID:AB_477193) and then mouse anti-GAPDH (1:10,000; Sigma-Aldrich, #CB1001 EMD MILLIPORE, RRID:AB_2107426). Membranes were then reacted with goat anti-mouse infrared (IR) dye-conjugated secondary antibody (Li-Cor antibody, #926-32210, RRID AB_621842) for 1 h at room temperature (RT). The membrane was scanned using an Odyssey® scanner, quantified using Fiji software, and analyzed with Prism 7.04 software (GraphPad).

### Transfection with miRNA mimic

Ctrl1 hNPCs were transfected with miRNA inhibitors using HiPerFect Transfection reagent (Qiagen, # 301705) according to the manufacturer’s instructions. The following inhibitors were used (purchased from Qiagen): hsa-miR-4273 miRCURY LNA miRNA mimic (339173); negative control A miRCURY LNA miRNA mimic (YM00479903). After 48 h, the medium was replaced with fresh medium, and cells were maintained for an additional 2 weeks before being fixed and stained.

### Quantitative (q)RT-PCR

For total RNA, RNA was extracted using a Quick-RNA™ MiniPrep kit (Zymo Research, #R1055), and each sample was reverse transcribed using a QuantiTect Reverse transcription kit (Qiagen, #205313). qRT-PCR reactions were performed with LightCycler 480 SYBR Green I MasterMix (Roche) in a LightCycler480II instrument (Roche). The primer sequence was designed using Primer Bank [36]. NRXN3: F- AGTGGTGGGCTTATCCTCTAC; R- CCCTGTTCTATGTGAAGCTGGA. S100β: F- TGGCCCTCATCGACGTTTTC; R- ATGTTCAAAGAACTCGTGGCA. The following primers were purchased from Qiagen: SOX2, NANOG, Lin28, β3-tubulin. Data were normalized to 18s rRNA expression, and analyzed with Prism 7.04 software (GraphPad).

miRNA was extracted using a mirVana™ miRNA Isolation Kit (Thermo Fisher, #AM1560), and each sample was reverse transcribed using miScript II RT Kit (Qiagen, #218161). qRT-PCR reactions were performed with miScript SYBR Green PCR Kit (Qiagen, #218075) in a LightCycler480II instrument (Roche). The following primers were purchased from Qiagen: miR-663b (MS00037884), miR-663 (MS00037247), miR-4273 (MS00021280). Data were normalized to RNU6-2 expression, and analyzed with Prism 7.04 software (GraphPad).

### Histological imaging and immunocytochemistry

For 2D cultures, hiPSC colonies, hNPCs, or neurons at 2 time-points of differentiation (1 and 3 months) were fixed with 4% PFA for 15 min and washed 3 times with PBS. Phase-contrast images of two-month-old 3D organoids were acquired on an EVOS cell imaging system (Thermo-Fisher Scientific) for size measurements. Two- to three-month-old cerebral organoids were fixed in 4% PFA at 4 °C overnight followed by serial incubation in 15% and 30% sucrose in PBS overnight. Fixed organoids were embedded in tissue freezing medium (TFM, General Data, Cincinnati, OH), and were flash frozen with isopentane and liquid nitrogen. Frozen organoids were sectioned in a cryostat using optimal cutting temperature (OCT) compound and then sectioned at 15-µm thickness. Cells or sections were blocked with 3% BSA and 0.3% Triton X-100 in PBS for 30 min. For synaptic staining, cultures were blocked with 3% BSA and 0.1% Saponin (wt/vol) in PBS. Cells/sections were incubated with primary antibody in blocking solution overnight at 4 °C and then washed with PBS. The appropriate Alexa Fluor (488, 555, 647) conjugated secondary antibodies were used at 1:1000, plus Hoechst 33342, Trihydrochloride, Trihydrate dye (1:1,000, Thermo-Fisher Scientific, #H3570) to visualize nuclei for 1 h at RT.

Primary antibodies and dilutions were as follows: Rabbit anti-NANOG (1:500, Cell signaling technologies, #4903s, RRID:AB_10559205); mouse anti-TRA1-60 (1:500, Cell Signaling Technologies, #4746s, RRID:AB_2119059); rabbit anti-SOX2 (1:500, Cell Signaling Technologies, #3579s, RRID:AB_2195767); mouse anti-nestin (1:250, Abcam, #ab22035, RRID:AB_446723); mouse anti-S100β (1:1000, Abcam, #ab11178, RRID:AB_297817); rabbit anti-GFAP (1:500, Agilent, #Z0334, RRID:AB_10013382); mouse anti-MAP2 (1:500, Sigma-Aldrich, #M4403, RRID:AB_477193); chicken anti-β3-tubulin (1:500, Abcam, #ab41489, RRID:AB_727049); guinea pig anti-VGLUT1 (1:250, Synaptic Systems, #135304, RRID:AB_887878); mouse anti-VGAT (1:250, Synaptic Systems, #131011, RRID:AB_887872); rabbit anti-synapsin I (1:500, Millipore, AB1543p, RRID:AB_90757); mouse anti-PSD95 (1:500, Invitrogen, MA1-045, RRID:AB_325399); rat anti-CTIP2 (1:150; Abcam, #ab18465, RRID:AB_2064130); rabbit anti-TBR2 (1:300; Abcam, #ab23345, RRID:AB_778267); rabbit anti-TBR1 (1:250, Abcam, #ab31940, RRID:AB_2200219); rabbit anti-GABA (1:500, Thermo Fisher Scientific Cat# PA5-32241, RRID:AB_2549714); rabbit anti-brain lipid binding protein (BLBP) (1:200, Millipore Cat# ABN14, RRID:AB_10000325). Coverslips were mounted on slides with fluorescent mounting medium (DAKO) and visualized with a Zeiss Axiovert (100M) epifluorescence microscope or ImageXpress automated high-content confocal microscopy (Molecular Devices). For synaptic staining of PSD95 and synapsin I, punctae were visualized using a Nikon A1 confocal microscope. Imaging conditions were identical for each set of stains (e.g., exposure time and laser power). Quantification of GFAP, MAP2, nestin, TBR2, TBR1, CTIP2, GABA, synapsin I, and PSD-95 were performed with Fiji software. The number of synapses was calculated as co-localized punctae of synapsin I/PSD-95 staining per neurite length identified with β3-tubulin with Fiji software. Quantification of S100β, β3-tubulin, VGLUT1, and VGAT were performed using MetaXpress (Molecular Devices). Quantification of immunostained cells was performed in a masked fashion, normalized to cell number (by Hoechst staining for cell nuclei), and compared with the same masking/thresholding settings.

### Luciferase gene reporter assays

Luciferase reporter assay was performed as previously described with some modifications [37]. Briefly, hNPCs were transfected with MEF2 luciferase reporter [12, 30, 38], which is expressed under the EF1α promoter, along with *Renilla* luciferase (expressed under CMV promoter) control vector using a Human Stem Cell Nucleofector Kit according to the manufacturer’s instructions (Lonza, VPH-5012). Cells were plated in a 96-well plate and terminally differentiated into neurons. Cells were harvested at various time points of differentiation over a period of 11 days and then analyzed using a Dual-Glo luciferase assay kit (Promega) following the manufacturer’s instructions. Firefly luciferase activity was normalized to *Renilla* luciferase activity.

### Bulk RNA-seq data acquisition, mapping, and analysis of 2D cultures

After 5 weeks in culture, hiPSC-derived neurons grown on mouse neonatal astrocytes were processed for analysis. The following control and patient samples were used for RNA sequencing: Control samples Ctrl1, Ctrl2 and Ctrl4, and patient mutations MHS-P1, MHS-P2, MHS-P3 and MHS-P4 (see Table S1). RNA was extracted using a Quick-RNA™ MiniPrep kit (Zymo Research, #R1055). RNA was quality checked before sequencing by RNA ScreenTape (Agilent tape station) and NanoDrop. All RNA samples had an RNA integrity number (RIN) >9. RNA libraries were prepared via Ribo depletion. Sequencing was performed on the Illumina HiSeq2500 platform in a 1 × 75 bp single end (SR) configuration in high output mode (V4 chemistry) with 40 million reads per sample. Reads were aligned to the latest human hg38 reference genome using the STAR spliced read aligner [39]. No read trimming or filtering was done with this data set because the quality distribution and variance appeared normal. Total counts of read-fragments aligned to known gene regions within the human hg38 RefSeq reference annotation were used as the basis for quantification of gene expression. Fragment counts were derived using HTS-seq program using hg38 Ensembl transcripts as model. Various QC analyses were conducted to assess the quality of the data and to identify potential outliers. Differentially-expressed genes (DEGs) were identified using three bioconductor packages, EdgeR, Limma+Voom and Limma, which were then considered and ranked based on adjusted p-values (FDR) of ≤0.1. Gene set enrichment analysis (GSEA) was performed using the top DEGs (determined by EdgeR). DEGs were selected based on several false discovery rate (FDR) Benjamini–Hochberg adjusted *p* values and simple *p* values. A total of 1029 genes were used for the enrichment analysis. We analyzed the expressed genome using the following software: PANTHER Overrepresentation Test (Released 20190711); GO Ontology database released 2019-02-02; Reference list: Homo sapiens (all genes in database). For the neuronal-relevant GO terms, most DEGs were further analyzed by qPCR to validate the results.

### ChIP-seq and qRT-PCR

ChIP assays were performed using the *ChIP-IT Express Kit* (Active Motif) according to the manufacturer’s instructions. For the ChIP-seq experiments, Ctrl1 was used, and the results were then validated on additional controls. Briefly, ~2 × 10^7^ hNPCs were used per ChIP experiment. The hNPCs were first dissociated with Accutase and crosslinked with 1% paraformaldehyde, and then the nuclei were sonicated 10 times for 20 s at maximum settings using a Misonix Sonicator (Misonix, Farmingdale, NY). Sheared chromatin was immunoprecipitated with 4 μg of a specific MEF2 antibody (#sc-313, Santa Cruz Biotechnology, RRID:AB_631920) or control IgG (Active Motif) antibody. At this early stage of development, NPCs express primarily the MEF2C paralog of the MEF2 transcription factor family [11], so predominantly MEF2C targets were detected. ChIP DNA samples were subjected to qPCR assay. We determined the enrichment of specific DNA sequences after ChIP using an EXPRESS SYBR® GreenER™ detection kit (Invitrogen) on a Mx3000P real-time PCR system (Stratagene). Levels of enrichment after ChIP were calculated using the comparative cycle threshold method (Invitrogen) after normalizing with IgG control, as we have previously described [40, 41]. ChIP DNA samples were also subjected to preparation for ChIP-Seq Library according to Illumina’s ChIP-Seq Sample prep kit. ChIP-seq samples were sequenced with an Illumina Genome Analyzer II system (San Diego, CA) according to the manufacturer’s instructions and aligned to the University of California, Santa Cruz (UCSC) *Homo sapiens* reference genome (hg18) using Bowtie. The aligned reads were processed with HOMER v2.6 software and visualized on the UCSC genome browser [42]. Peaks were predicted and annotated with HOMER v2.6 software (Table S2).

### Single cell (sc)RNA-seq of cerebral organoids

After 3 months in culture, individual cerebral organoids were dissociated into a single-cell suspension as previously described [43] using the Worthington Papain Dissociation System kit (Worthington Biochemical). In brief, dissociated organoids were resuspended in ice-cold NbActiv-1 containing 1% BSA at a concentration of 3000 cells/μl, and labeled with CellPlex cell multiplexing oligonucleotides conjugated to a lipid (10x Genomics). Cells were loaded onto a Chromium Single Cell 3′ Chip (10x Genomics) and processed through the Chromium controller to generate single-cell gel beads in emulsion (GEMs). scRNA-seq libraries were prepared from captured cells using standard protocol and libraries sequenced on a NextSeq 2000 instrument (Illumina).

CellRanger (10x Genomics) was used to generate digital expression matrices from the FASTQ files obtained from the Illumina sequencing runs. The digital expression matrices for cells that passed rigorous quality control were then input into the Seurat R package (v3.0) to generate Seurat objects for comprehensive downstream analyses and visualization. The following Seurat functions were used in the Seurat pre-processing pipeline: HTODemux was used to demultiplex the samples mixed in the same 10x run; NormalizeData, ScaleData were used for calculating the comparable expression values; FindVariableFeatures was used to include the variable genes that contribute to the overall similarity/variability of cellular transcriptomic profiles; RunPCA, FindNeighbors, FindClusters, RunTSNE, and RunUMAP were used to calculate the dimension-reduction coordinates for visualization and to perform unsupervised clustering. In downstream analyses, we used Uniform Manifold Approximation and Projection (UMAP) coordinates to visualize the layout of the cells. We applied the SuperCT algorithm [44] to scRNA-seq and cell-type annotations from the Pașca laboratory [43, 45] as the training dataset in order to generate our cell-type classification model. We also used canonical markers that were enriched in the unsupervised clusters in order to cross-validate the identity of the clusters. Finally, the summary statistics of these annotated clusters were used for population comparisons. After the initial cell-type classification and annotation, we performed additional unsupervised subclustering analysis using the Seurat function FindClusters on the radial glial cell (RGC) and GABAergic interneuron populations identified by the previous annotation. Prior to further analysis, we filtered out cells with only a low number of genes detected. The resolution for the clustering was set at 0.4. The resulting subclusters were then analyzed to investigate their features as described in the text.

### Differential gene expression (DEG) analysis of scRNA-seq data from cerebral organoids

DEG analyses in specific cell populations were conducted on the cell types determined by SuperCT using the Seurat FindAllMarkers function. Accordingly, we first used the subset of the target cell types. The Seurat object of this subset of cells was then used to find markers, and grouped by organoid types (isogenic wild-type [WT] or mutant). Output genes of FindAllMarkers were considered differential genes.

To compare the DEG for NRXN3, for example, we used the TPM (number of transcripts of a specific gene per million total molecules, counted by barcodes) to measure gene expression in a group of cells of a specific type. A two-tailed paired Student’s *t-*test of NRXN3 expression data from 3 mutant vs. 3 WT samples showed statistically increased expression of NRXN3 in hiPSC-derived GABAergic neurons (see manuscript text for details).

### Rank-rank hypergeometric overlap (RRHO) analysis

To compare gene expression profiles between bulk RNA-seq and scRNA-seq datasets, we performed a RRHO analysis. First, we calculated pseudo-bulk expression values from the scRNA-seq data. For each isogenic cell group, we summarized the unique molecular identifiers (UMIs) of specific genes and the total UMIs from the scRNA-seq UMI matrix. Pseudo-bulk expression values were calculated as the ratio of gene-specific UMIs to total UMIs in each cell group. We then used the RRHO package in R to perform the rank-rank hypergeometric overlap test, comparing the differentially expressed genes between isogenic types from both scRNA-seq and bulk RNA-seq studies.

### Patch-clamp electrophysiology and pharmacology experiments

Whole-cell recordings were performed with patch electrodes of 3 to 5 MΩ resistance. To analyze voltage-gated currents, pipettes were filled with an internal solution composed of (in mM): K-gluconate, 120; KCl, 5; MgCl_2_, 2; HEPES, 10; EGTA; 10; Mg-ATP, 4; pH 7.4 and mOsm 290. The external solution was composed of Ca^2+^ and Mg^2+^-free Hank’s Balanced Salt Solution (HBSS; GIBCO) to which were added: CaCl_2_, 2 mM; HEPES, 10 mM; and glycine, 20 mM. The presence of sodium current was established by inhibition with 1 µM TTX. Potassium currents were confirmed by their inhibition with 20 mM TEA chloride (Tocris) and 5 mM 4AP (TCI). Recordings were performed using a MultiClamp 700B amplifier (Molecular Devices) at a data sampling frequency of 2 kHz with an analog-to-digital converter, Digidata 1440 A (Molecular Devices). Voltage-clamp and current-clamp protocols were applied using Clampex v10.6 (Molecular Devices). Preliminary analysis and offline filtering at 500 Hz were achieved using Clampfit v10.6 (Molecular Devices).

For recording evoked action potentials under current clamp, we hyperpolarized the neurons to relieve depolarization block. This was necessary because hiPSC-derived neurons are typically depolarized in culture, as they are relatively immature [46, 47] because potassium channels underlying the inward rectifier K^+^ current (Kir) develop late and are known to regulate the resting membrane potential [48].

Agonist-induced currents (glutamate- and GABA-evoked) were recorded in voltage clamp mode at a holding potential of –70 mV in the nominal absence of extracellular Mg^2+^ and in the presence of 20 μM glycine (Sigma) and TTX (1 μM, Hello Bio). Agonist was applied via a rapid gravity-flow, local superfusion system. The internal solution for these recordings was (in mM): CsCl, 135; MgCl_2_, 2; HEPES, 10; EGTA; 1; Mg-ATP, 4; pH 7.4 and mOsm 290.

Evoked postsynaptic currents were induced by brief (1 ms) unipolar current pulses (0.1–0.9 mA) from an external stimulator (A365, WPI) using a stimulating electrode (CBAEC75, FHC) positioned at a distance of 100–150 μm from the cell soma [49]. The internal solution for these recordings was composed of (in mM): K-gluconate, 120; KCl, 5; MgCl_2_, 2; HEPES, 10; EGTA; 10; Mg-ATP, 4; pH 7.4 and mOsm 290. The external solution comprised Ca^2+^ and Mg^2+^-free Hank’s Balanced Salt Solution (HBSS; GIBCO) to which were added the following: MgCl_2_, 2 mM; CaCl_2_, 2 mM; HEPES, 10 mM; and glycine, 20 mM.

Spontaneous postsynaptic currents (sPSCs) were recorded at a holding potential of –70 mV and 0 mV in gap-free mode as described in previously [32]. The internal solution contained (in mM): K-gluconate, 120; KCl, 5; MgCl_2_, 2; HEPES, 10; EGTA; 10; Mg-ATP, 4; pH 7.4 and mOsm 290. Miniature excitatory postsynaptic currents (mEPSCs) and miniature inhibitory postsynaptic currents (mIPSCs) were recorded at –70 mV and 0 mV, respectively, at 21 °C after equilibrium in TTX (1 µM) for at least 20 min. The internal solution used for recording mEPSCs and mIPSCs was comprised of (in mM): Cs-gluconate 130; CsCl 5; MgCl_2_, 2; HEPES, 10; EGTA; 1; Mg-ATP, 4; pH 7.4 and mOsm 290. Mini Analysis software (Synaptosoft, Fort Lee, NJ) was used to calculate the frequency and amplitude of spontaneous synaptic events.

### Calcium imaging

For monitoring the relative change in intracellular Ca^2+^ concentration vs. time, Fluo-4 (Fluo 4 AM/Fluo 4 direct, Life Technologies) was used according to the manufacturer’s instructions and a previously published protocol [50]. To monitor Fluo-4 fluorescence changes, randomly selected fields of comparable cell density were excited at 480 nm with an exposure time of 30 ms, imaged using a 40X oil objective at 33 frames per second, and analyzed with MetaMorph software (Molecular Devices). Changes in intracellular Ca^2+^ from baseline (F_0_) to peak fluorescence (ΔF) were expressed as fractional changes above baseline (ΔF/F_0_). Events with ΔF/F rise times of <200 ms were considered neuronal transients. Neurons were distinguished from astrocytes morphologically, as verified with cell-type specific antibody staining in previously published experiments [32]. Calcium imaging movies using Fluo-4 were prepared at 500 frames per second using ImageJ (NIH). We attempted to enrich for extrasynaptic NMDA-type glutamate receptor (eNMDAR)-mediated Ca^2+^ responses over synaptic responses using a previously published protocol with bicuculline and MK-801, as delineated above [51], but see the caveats for this procedure discussed in the legend to Fig. S8.

### Multielectrode array (MEA) recording

MEA recordings were obtained as previously described [50]. For 2D cultures, cells were plated on CytoView 12-well plates (Axion Biosystems) coated with 0.1% polyethyleneimine (PEI) and 10 μg/ml laminin. For 3D cultures, single 6-week-old cerebral organoids were plated on CytoView 12-well plates (Axion Biosystems) coated with 0.1% PEI and 10 μg/ml laminin, as we previously described [32, 50]. Based upon pilot experiments performed to determine when neuronal properties developed, we recorded from 2D cultures ~5–6 weeks after neuronal differentiation from hNPCs, and from cerebral organoids at 3–4 months of development. Recordings were performed on a Maestro MEA (Axion Biosystems) using the “neural broadband analog mode” setting with a sampling frequency of 12.5 kHz in control conditions and after drug treatment at 37 °C and analyzed with Axion Biosystems Maestro Axis software (version 2.4.2). For calculating firing frequency, an electrode displaying >5 spikes per min was considered active. A network burst was defined as a minimum of 25 electrodes displaying >10 spikes per electrode at <100 ms inter-spike interval. Network burst frequency was calculated as total number of network bursts recorded per second during the analysis window expressed in Hz. Synchronous firing was determined by analyzing a 20 ms synchrony window, which is the time window around zero used for computing the area under the cross-correlation curve. Analysis of well-wide, pooled interelectrode cross-correlation gives the synchrony index, defined as a unitless measure of synchrony between 0 and 1 such that values closer to 1 indicate higher synchrony.

## Quantification and statistical analysis

### Statistical analysis

For each experiment, we used at least 3 independent sets of cultures from separate differentiations or 3 independent groups of cerebral organoids from separate differentiations. Sample size was determined from prior power analyses based on prior data obtained in our laboratory. All data were acquired by an investigator blinded to the sample groups. No data was excluded from analysis. Data are presented as mean ± SEM. Statistical analyses were performed using GraphPad Prism software. Statistical significance was determined by a two-tailed unpaired Student’s *t*-test for single comparisons, by ANOVA followed by a post hoc Dunnett’s test for multiple comparisons with single Ctrl, or by a Sidak’s test corrected for multiple comparisons with multiple Ctrls and between selected pairs. For non-parametric data such as percentage change in calcium event frequency, we used a Kruskal–Wallis test followed by a post hoc Dunn’s test for multiple comparisons and Mann–Whitney U test for single comparisons. For analysis of cumulative distributions, we used the Kolmogorov–Smirnov test performed with Mini Analysis software (Synapstosoft, Fort Lee, NJ). Data with *p* values < 0.05 were considered to be statistically significant. In general, the variance was similar between groups being compared. No samples or data were excluded from the analysis. In graphs with sample size <26, individual datapoints are shown, as more points cannot be resolved sufficiently. Exact *p* values for all comparisons made in the main figures and supplementary figures are listed in a separate file (Supplementary Information file 1).

## Results

### Altered differentiation of MHS hiPSC-derived neurons in 2D culture

To study the molecular mechanisms of MHS in human context, we generated hiPSC lines from dermal fibroblasts of 3 patients (MHS-P1, MHS-P3, MHS-P4) bearing a microdeletion or point mutation in *MEF2C*. As controls, we used sex-matched samples of similar ages consisting of well-characterized hiPSC lines (Ctrl1-3) [52]. Additionally, using CRISPR/Cas9, we generated an hiPSC line with a microdeletion in *MEF2C* (designated MHS-P2~) that could be compared to its cognate isogenic control (Ctrl1~; see Table S1). In total, we analyzed 4 hiPSC lines with mutations in MEF2C and 3 control lines. All hiPSC lines were characterized and found to be karyotypically normal, positive for pluripotency markers, and capable of differentiating into all germ layers (Fig. S1). We differentiated the hiPSCs into cerebrocortical neurons using a dual-SMAD inhibition protocol [32, 33], Both MHS patient and Ctrl hiPSCs efficiently generated hNPCs, as detected by immunofluorescent labeling with nestin and SOX2 (Fig. S2A). The cells were subsequently differentiated into cerebrocortical neurons (hiPSC-neurons) expressing pan-neuronal markers, including β3-tubulin and microtubule associated protein 2 (MAP2). Our differentiation protocol yielded both excitatory and inhibitory neurons, as well as astrocytes [32].

**Fig. 1:**
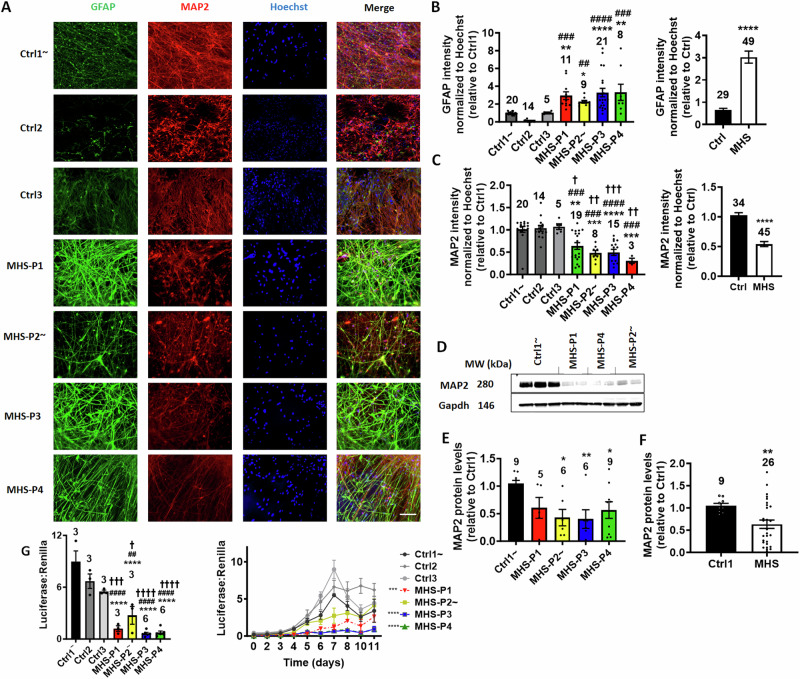
MHS hiPSCs in 2D cultures generate more astrocytes and fewer neurons, but with increased spontaneous activity compared to controls (Ctrls). **A** Representative images of GFAP, MAP2, Hoechst (to label cell nuclei), and merged images in 4-week-old hiPSC-derived cultures. Patient-relevant mutation-bearing hiPSCs abbreviated MHS-P1 through P4. The genetic background of MHS-P2 is isogenic to Ctrl1. Scale bar, 100 µm. **B** Quantification of GFAP intensity for each MHS line normalized to Hoechst and relative to Ctrl1 (*at left*). Grouped analysis of GFAP expression for Ctrl vs. all MHS patients (*at right*). **C** Quantification of MAP2 intensity for each MHS line normalized to Hoechst and relative to Ctrl1 (*at left*). Grouped analysis of MAP2 expression for Ctrl vs. all MHS patients (*at right*). **D** Representative immunoblot of MAP2 expression with GAPDH as loading control. **E** Quantification of MAP2 protein expression for each MHS line. **F** Grouped analysis of MAP2 expression showing a decrease in protein levels in MHS lines vs. Ctrl1. **G** Comparison of MEF2 luciferase reporter gene activity normalized to Renilla at day 7 of differentiation of MHS hiPSCs compared to controls (*at left*), and at various time points of neuronal differentiation for each MHS line vs. Ctrls (*at right*). Statistical analysis was performed on area under the curve (AUC). Values are mean ± SEM. Sample size listed above bars (number of fields for immunofluorescence panels in ≥3 independent experiments). Individual datapoints shown on bar graphs wherever possible in this and subsequent figures (for sample sizes <25). *^,#,†^*p* < 0.05, **^,##,††^*p* < 0.01, ***^, ###, †††^*p* < 0.001, ****^,####, ††††^*p* < 0.0001 by Student’s *t*-test for pairwise comparisons or ANOVA with Dunnett’s post-hoc test for multiple comparisons to single Ctrl and with Sidak’s test for comparison to multiple Ctrls; comparison to Ctrl1 (*), Ctrl2 (^#^), Ctrl3 (^†^) (see Methods). Ctrl1 and its isogenic line MHS-P2 designated by (~) in this and subsequent figures.

To begin to assess the differentiation capacity of the various hiPSC lines, we analyzed neuronal- and astrocyte-selective markers (MAP2 and glial acidic fibrillary protein (GFAP) or S100β, respectively) after 1 month of differentiation from hNPCs. Compared to Ctrls, we discovered that hNPCs bearing an MHS-related mutation generated more astrocytes, as detected by GFAP and S100β, and fewer MAP2-positive neurons (Figs. 1A–C, 1F, S2B and S2C). We also found lower levels of MAP2 protein in hiPSCs bearing MHS-relevant mutations (MHS hiPSC-neurons) compared to Ctrl1, as detected by immunoblot (Fig. 1D, E). MAP2 levels were similarly lower in 3-month-old neuronal cultures (Fig. S2D, S2E), indicating that this effect does not appear only at early stages of development, and persists. It should also be noted that early in development there can be some overlap of MAP2 and GFAP markers [53], presumably because GFAP labels RGCs (which can become neurons) in addition to astrocytes. That is why we show the more specific astrocyte label S100β in Fig. S2.

Additionally, as cells were transitioning from hNPCs to neurons during the first week of terminal differentiation, we performed MEF2-reporter gene assays to begin to assess whether MEF2C participates in the early stages of neuronal differentiation and maturation. We found that the human neurons generated from MHS hiPSC-neurons had lower MEF2 luciferase activity consistent with haploinsufficiency, with the most significantly different values at day 7 of differentiation (Fig. 1G).

### Transcriptomic effects on MHS hNPCs drive the astrocytic phenotype and limit neurogenesis

**Fig. 2:**
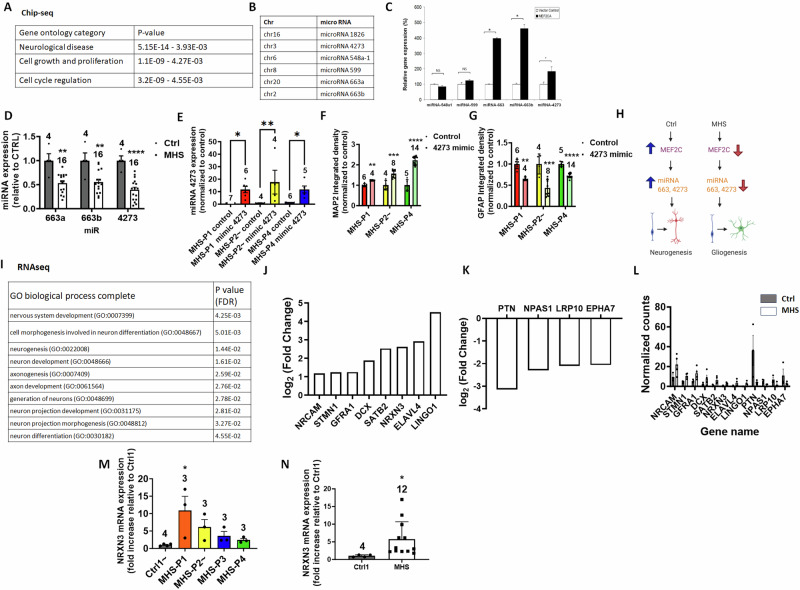
ChIP-seq and RNA-seq analyses show MEF2C effects on hiPSC-derived cell types in 2D cultures. **A** Top gene ontology (GO) terms for hits found in ChIP-seq analysis of the 198 MEF2C binding targets in hNPCs. **B** List of miRNAs identified that contain binding sites for MEF2C. **C** Relative gene expression levels of miRNA identified by ChIP-seq in control hNPCs and hNPCs expressing constitutively active MEF2 containing a VP16 transactivation domain (MEF2CA). **D** Relative gene expression of miRNA in Ctrl and MHS patient hiPSC-derived cells after 2 weeks in culture. **E** Relative gene expression of miRNA in MHS patient hiPSC-derived cells exposed to ‘miR-4273 mimic’ or control miR after 2 weeks in culture. **F** MAP2 neuronal marker expression in MHS hiPSC-derived cells expressing ‘miRNA mimic’ compared to non-target control mimic after 2 weeks in culture. **G** GFAP astrocytic marker expression in MHS hiPSC-derived cells expressing ‘miRNA mimic’ compared to non-target control mimic after 2 weeks in culture. **H** Schematic diagram of miRNA effect on neurogenesis and gliogenesis. **I** Top GO biological process terms based on differentially-expressed genes (DEGs) by RNA-seq after 5 weeks in culture in MHS patient hiPSC-neurons vs. Ctrl showing neuronally-enriched pathways. **J** Top DEGs from RNA-seq showing higher expression in MHS hiPSC-neurons vs. Ctrl. **K** Top DEGs from RNA-seq showing lower expression in MHS hiPSC-neurons vs. Ctrl. **L** Bar Plot of normalized counts for the various genes acquired from RNA seq data on control lines (Ctrl1, 2 and 4) and on patient lines (MHSP1-4). **M** NRXN3 mRNA expression in each MHS patient vs. Ctrl. **N** NRXN3 mRNA expression in all MHS patients combined vs. Ctrl. Data are mean + SEM. Sample sizes (n) are listed above bars from at least 3 independent experiments. *p < 0.05, **p < 0.01 by ANOVA with Dunnett’s post-hoc test for multiple comparisons or by two-tailed Student’s *t*-test for single comparisons.

To understand mechanistically how MEF2C affects differentiation into neurons, we performed chromatin immunoprecipitation-sequencing (ChIP-seq) analysis on control hNPCs for MEF2C binding sites, using methods we have detailed previously [40, 41] (Figs. 2A, B, and S5). We found 198 such targets (Table S2), many of which were enriched for gene ontology (GO) terms related to neurological diseases, cell growth, and proliferation (Fig. 2A). Interestingly, five of these MEF2C targets encoded microRNAs (miRNAs) thought to be involved in neurogenesis vs. gliogenesis [54, 55], so we focused on these (Fig. 2B). We first validated the miRNA results using hNPCs that express a constitutively active form of MEF2C [37, 56, 57] and found that three miRNAs, miR-663a, miR-663b and miR-4273, all of which have binding sites on astrocytic-expressed mRNAs [58], manifested higher expression when MEF2C was active (Fig. 2C). We then found that patient MHS cells had lower expression of all three of these miRNAs **(**Fig. 2D). When we increased the expression of miR-4273 in MHS hNPCs using a miR mimic to rescue the effect of haploinsufficiency, we found that the cells differentiated into a greater percentage of neurons and fewer astrocytes, as detected by MAP2 and GFAP staining, respectively (Figs. 2E–G, and S5D). Thus, by using miR-4273 we were able to reverse the ratio of astrocytes to neurons in 2D cultures. Conversely, inhibition of miR-663a and miR-4273 in control cells using commercially-available specific inhibitors (see Methods) recapitulated disease phenotypes, including decreased MAP2 integrated density (a neuronal marker indicative of decreased neurogenesis) and increased S100β integrated density (indicative of increased gliogenesis) (Fig. S5E, S5F). Taken together, these findings are consistent with the notion that suppression of these miRNAs in MHS patient cells contributes to progenitor differentiation into fewer neurons and more astrocytes very early in development (Fig. 2H).

Next, using methods we have described [59], we performed RNA-sequencing (RNA-seq) 5 weeks after neuronal induction to detect changes in gene expression between MHS and Ctrl cells in 2D culture after differentiation on a layer of mouse primary astrocytes. Previously, we had found that MEF2C predominantly targets neuronally-restricted gene promoters starting at the hNPC stage [56]. Accordingly, when we analyzed gene enrichment by GO terms, we encountered several terms related to neuronal differentiation and function (Fig. 2I). These findings are in agreement with prior transcriptomic analysis of MEF2-regulated genes at the hNPC stage [56]. Among the genes found to be enriched in our dataset, several are known to be predominantly expressed in neurons, as validated in neuronal cultures (Fig. 2J–L). Neurexin3 (*NRXN3*) was of particular interest because of its known involvement in neuronal development and function [60]. Interestingly, neurexins have been associated with ASD, and high or low expression can be detrimental, pointing to a bell-curve effect as found for MEF2C [61], with an optimal dose of neurexin critical for normal brain development [62]. Furthermore, region- and context-specific effects of alterations in NRXN3 have been demonstrated [60]. Along these lines, we found that NRXN3 was increased in MHS patient hiPSC-neurons compared to Ctrl in our RNA-seq data from 2D cultures (Fig. 2M, N). Collectively, both our ChIP-seq and RNA-seq results support the notion that gene expression in our MHS hiPSC-derived cells correlates with pathways known to be involved in dysfunctional neurogenesis, increased gliogenesis, and aberrant neuronal differentiation, which we therefore investigated further.

### MHS hiPSC-neurons in 2D culture show enhanced spontaneous action potential frequency and glutamate-evoked currents, but decreased GABA-evoked currents

**Fig. 3:**
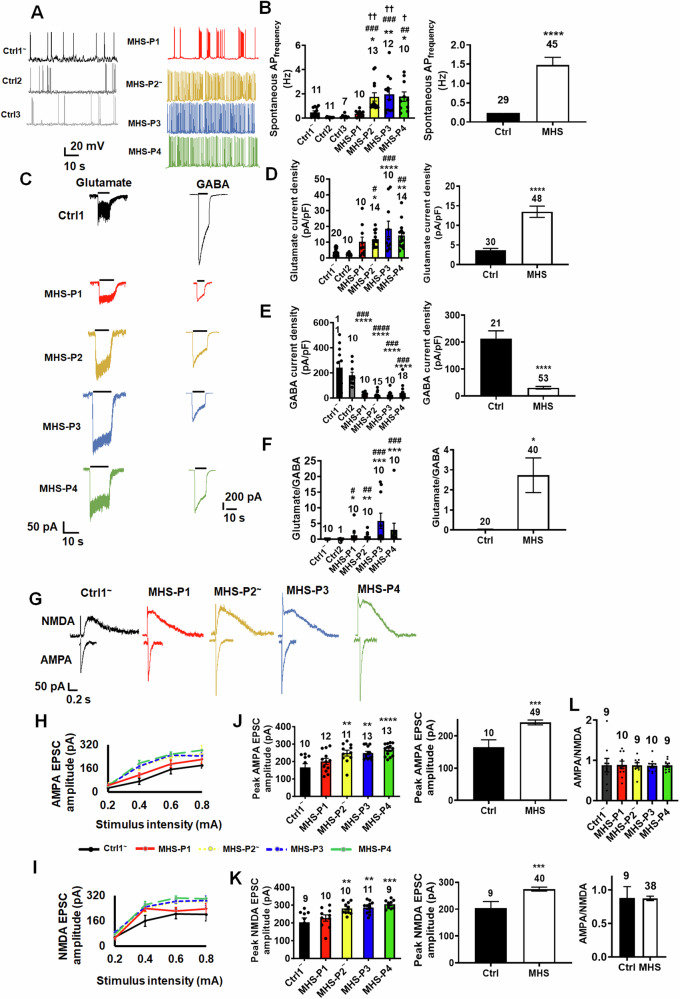
MHS hiPSC-derived cerebrocortical neurons show increased excitation and decreased inhibition in 2D cultures. **A** Recordings of spontaneous action potential (sAP) at resting membrane potential (RMP). **B** Quantification of sAP frequency in neurons from each MHS patient (P1-P4) compared to each control (Ctrl1, Ctrl2, Ctrl3) *on left*; grouped analysis *on right*. **C** Representative traces of glutamate- and GABA-evoked currents (each at 100 µM). **D**, **E** Quantification of glutamate and GABA current density. **F** Ratio of glutamate to GABA current densities. **G** Representative patch-clamp recordings of evoked AMPAR-EPSCs at holding potential (V_h_ = −70 mV) and NMDAR-EPSCs (V_h_ = +60 mV). **H**, **I** Input-output curves of evoked AMPAR-EPSCs and NMDAR-EPSCs. **J**, **K** Quantification of peak current amplitude of evoked AMPAR-EPSCs and NMDAR-EPSCs from each patient. **L** Quantification of ratio of peak AMPA/NMDA EPSCs from individual neurons for Ctrl and each MHS patient (*above*); grouped analysis (*below)*. Data are mean ± SEM. Number of neuronal recordings (n) listed above bars from at least 4 experiments in each case. *^,#^*p* < 0.05, **^,##^*p* < 0.01; ***^,###^*p* < 0.001, ****^,####^*p* < 0.0001 by ANOVA for multiple comparisons with Sidak’s post-hoc test in **B**, **C**, **D** or Dunnett’s post-hoc test in H and I; comparison to Ctrl1 (*), Ctrl2 (^#^), Ctrl3 (^†^) (see Methods).

Electrophysiologically, we found that the decrease in MEF2 reporter gene activity correlated with hyperexcitability at both the single neuron and network level; we observed this effect within 5 weeks of neuronal differentiation in culture. For example, MHS hiPSC-neurons in 2D culture showed an ~2-fold increase in spontaneous action potential (sAP) frequency compared to Ctrl hiPSC-neurons (Fig. 3A, B). Note that these hiPSC-neurons were differentiated on mouse primary astrocytes to support development of synapses and functional neuronal network activity. A statistically significant increase in sAPs was encountered for each patient mutation except MHS-P1, which manifested an increase compared to Ctrl2 and Ctrl3 but not Ctrl1 (Fig. 3A, B). Note that sodium currents (evidenced by inhibition with tetrodotoxin (TTX)) and potassium currents (inhibited by 4-aminopyridine (4AP) and tetraethylammonium (TEA)), evoked-action potential frequency, and other action potential parameters, including firing threshold, height and half-width, did not show any significant differences between MHS and Ctrl hiPSC-neurons (Figs. S3, S4A, S4B; and Table S3).

Since both MEF2C and NRXN3 are particularly important for the development of synapses [11, 16, 29, 30, 60, 63], we next sought to understand if MHS hiPSC-neurons exhibit differential excitatory and inhibitory activity. We performed patch-clamp electrophysiological recordings on neurons plated on primary mouse astrocytes to enhance synaptic interactions [64]. Initially, we studied ligand-gated channels. During whole-cell recordings we found the current density elicited by exogenous glutamate application to be 2–4-fold greater in MHS hiPSC-neurons than Ctrl (Fig. 3C, D). In contrast, we found significantly decreased GABA-evoked current density in MHS hiPSC-neurons (Fig. 3C, E). Taken together, this resulted in a robust increase in the glutamate to GABA current ratio (Fig. 3F).

Next, we monitored evoked excitatory synaptic activity. Compared to control hiPSC-neurons, we found a statistically significant increase for MHS-P2-4 (with a similar trend for MHS-P1) in the NMDAR-mediated component of excitatory postsynaptic currents (NMDAR-EPSCs), monitored at +60 mV to ensure removal of Mg^2+^ block by depolarization. There was also an increase in the AMPA receptor-mediated component (AMPAR-EPSCs), measured at –70 mV to ensure separation from the NMDAR-mediated component by blocking NMDAR-operated channels via hyperpolarization-induced Mg^2+^ block (Fig. 3G–K). There was no significant difference in the AMPA:NMDA ratio (Fig. 3L). Too few evoked inhibitory synaptic currents were observed under our conditions to allow quantification, possibly because of prior findings from the Fishell group showing that MEF2C also plays a role in generating parvalbumin (PV)-positive GABAergic interneurons and as our group has reported in MHS-model mice [30, 65]. Other electrophysiological parameters, including resting membrane potential, membrane resistance, cell capacitance, sodium- and potassium-current density, were similar among the MHS and Ctrl hiPSC-neurons (Table S3).

### MHS hiPSC-neurons in 2D culture exhibit disrupted spontaneous synaptic transmission

**Fig. 4:**
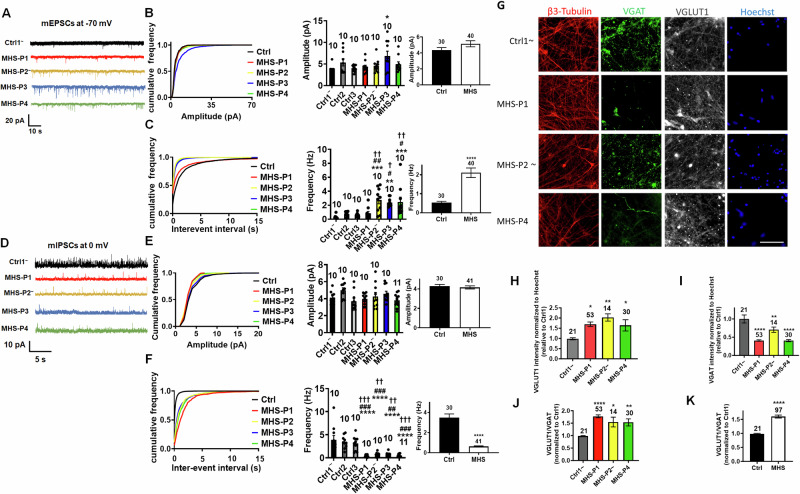
MHS hiPSC-derived cerebrocortical neurons exhibit disrupted synaptic transmission in 2D cultures. **A** Representative mEPSCs recorded at –70 mV in the presence of 1 µM TTX from Ctrl1 and MHS hiPSC-neurons in culture for 5 weeks. **B**, **C** Cumulative probability and quantification of mean mEPSC amplitude and interevent interval (inversely related to frequency). Cumulative probability of MHS mEPSC interevent interval was significantly decreased compared to Ctrl (*p* < 0.0001 by Kolmogorov–Smirnov test). **D** Representative mIPSCs recorded at 0 mV. **E**, **F** Cumulative probability and quantification of mean mIPSC amplitude and interevent interval. Cumulative probability of MHS mIPSC interevent interval was significantly increased compared to Ctrl (*p* < 0.0001 by Kolmogorov–Smirnov test). For bar graphs in **B**, **C**, **E** and **F**, responses of each MHS patient’s neurons vs. each control shown *on left*, with grouped MHS patient vs. controls shown *on right*. **G** Representative images of β3-tubulin, VGLUT1, VGAT, and Hoechst in Ctrl1 and MHS hiPSC-neurons. Scale bar, 100 µm. **H** Quantification of VGLUT1 in various MHS hiPSC-neurons compared to Ctrl1. **I** Quantification of VGAT in various MHS patient neurons compared to Ctrl1. **J** Quantification of the VGLUT1/VGAT ratio in various MHS patients. **K** Summary of VGLUT1/VGAT in Ctrl1 vs. MHS as a group. Data are mean + SEM. Number of neuronal recordings or imaged fields (n) listed above bars from at least 4 experiments in each case. *^,#,†^*p* < 0.05, **^,##,††^*p* < 0.01, ***^,###,†††^*p* < 0.001, ****^,####,††††^*p* < 0.0001 by ANOVA with Sidak’s post-hoc test in **B**, **C**, **E** and **F** for comparison to Ctrl1 (*), Ctrl2 (^#^), Ctrl3 (^†^); Dunnett’s post-hoc test for multiple comparisons with single Ctrl (see Methods).

To further examine differences between excitatory and inhibitory signaling, we next focused on spontaneous synaptic activity. We observed a statistically significant increase in the frequency of spontaneous (s)EPSCs in all MHS hiPSC-neurons except MHS-P1, which manifested a trend in this direction (Fig. S6A, S6C). Additionally, sEPSCs recorded from MHS-P3 and -4 exhibited increased amplitude compared to Ctrl hiPSC-neurons (Fig. S6A, S6B). Concerning spontaneous miniature EPSCs (mEPSCs), we found an increase in frequency with no change in amplitude for most MHS hiPSC-neurons (Fig. 4A–C). MHS-P3, however, displayed a small increase in mEPSC amplitude. In contrast to increased excitatory synaptic transmission, we observed a significant decrease in the frequency of spontaneous and miniature inhibitory postsynaptic currents (sIPSCs and mIPSCs) in MHS hiPSC-neurons compared to Ctrl, without a significant change in amplitude (Figs. 4D–F and S6D–S6F). Notably, we had previously reported similar findings in a *MEF2C* heterozygous mouse model of MHS that displays increased excitatory synaptic transmission and decreased inhibitory synaptic transmission [30].

The increase in mEPSC and decrease in mIPSC frequencies may reflect alterations in presynaptic mechanisms, such as quantal release, or a change in excitatory and inhibitory synapse numbers, or both types of effects. To begin to distinguish between these possibilities, we performed a series of immunocytochemical experiments to monitor synaptic properties. Initially, we measured the total number of excitatory synapses by counting co-localized punctae of presynaptic synapsin I and postsynaptic PSD-95 and found that MHS hiPSC-neurons displayed a moderate, statistically significant decrease in the total number of synapses compared to Ctrl (Fig. S7). Next, we monitored presynaptic vesicle proteins and observed an increase in vesicular glutamate transporter (VGLUT1) staining but a decrease in vesicular GABA transporter (VGAT), resulting in an increase in the ratio of VGLUT1 to VGAT in MHS hiPSC-neurons compared to Ctrl (Fig. 4G–K). These changes are similar to those we had observed in *MEF2C* heterozygous mice [30] and consistent with a presynaptic effect on neurotransmission. The findings on excitatory presynaptic terminals may reflect in part the known action of NRXN3 on VGLUT1 expression and clustering [60, 62, 63]. Collectively, our results are consistent with the notion that despite a decrease in the total number of excitatory synapses, MHS hiPSC-neurons may exhibit enhanced presynaptic release, resulting in an overall increase in excitatory neurotransmission. Moreover, in addition to its role in neurogenesis and synaptogenesis during development [11], MEF2C is also known to play a critical role in excitatory synaptic pruning once synapses have been formed [14, 66–68]. Hence, the relative deficit in MEF2C transcriptional activity in MHS hiPSC-neurons compared to Ctrl could also contribute to the increase in mEPSC frequency as a result of decreased excitatory synaptic pruning of MHS hiPSC-neurons as they mature. In contrast to excitatory neurotransmission, we found that inhibitory transmission is relatively depressed by both electrophysiological and histological parameters in MHS hiPSC-neurons. Taken together, the increase in excitation and decrease in inhibition that we observed in MHS hiPSC-neurons may lead to hyperexcitability in the neural network, thus contributing to ASD-like pathophysiology in MHS patients. Therefore, in the next series of experiments we investigated the properties of neural network activity in MHS vs. Ctrl using hiPSC-derived 2D cultures and 3D cerebral organoids.

### MHS hiPSC-neurons in 2D culture exhibit aberrantly enhanced neural network activity reflected in spontaneous calcium transients and multielectrode array recordings that are normalized by the NMDAR antagonist NitroSynapsin

**Fig. 5:**
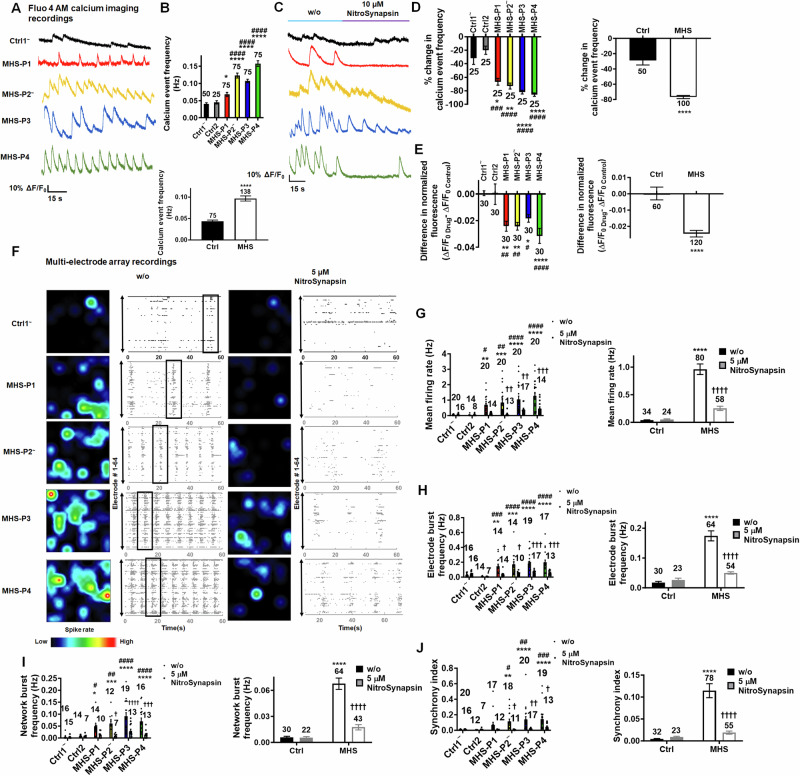
NitroSynapsin normalizes spontaneous calcium transients and neural network activity in MHS hiPSC-derived cerebrocortical neurons in 2D cultures. **A** Spontaneous neuronal calcium transients recorded from individual Ctrl1 and MHS hiPSC-neurons loaded with Fluo-4 AM. **B** Quantification of Ca^2+^ transient frequency for events with rise times <200 ms (individual Ctrl1 and MHS hiPSC-neurons responses in *upper panel*, and grouped responses in *lower panel*). **C** Representative calcium traces showing decrease in spontaneous calcium transient frequency after application of 10 µM NitroSynapsin. **D** Quantification of calcium transient frequency before and after application of NitroSynapsin. **E** Quantification of difference in normalized fluorescence (ΔF/F_0Drug_–ΔF/F_0Control_) as area under the curve (AUC) in response to NitroSynapsin. **F** Representative heat maps and raster plots of MEA recordings from Ctrl and MHS hiPSC neurons before (w/o) and after treatment with 5 µM NitroSynapsin. Boxes outline examples of network bursts. **G**–**J** Quantification of MEA recordings by mean firing rate, electrode burst frequency (representing bursting of individual neurons), network burst frequency (representing bursting of the entire neural network), and synchronous firing. Data are mean ± SEM. Sample size listed above bars represents number of cells (n) analyzed in 5–10 independent experiments. In **D**, **E**, **G**, **H**, **I** and **J**, responses of each MHS patient’s 2D neurons vs. each control shown *on left*, with grouped MHS patient vs. controls shown *on right*. *^,#,†^*p* < 0.05, **^,##,††^*p* < 0.01, ^***, ###, †††^*p* < 0.001, ****^,####, ††††^*p* < 0.0001 by ANOVA by Sidak’s post-hoc test for comparison to Ctrl1 (*) or to Ctrl2 (^#^), or within a group (^†^) between NitroSynapsin treatment vs. without (w/o) treatment; for panel D, comparison was made by non-parametric Kruskal–Wallis test (see Methods).

Initially, to investigate neural network activity in a population of neurons simultaneously, we monitored Ca^2+^ transients in 2D cultures. MHS hiPSC-neuronal cell bodies displayed an increase in spontaneous Ca^2+^ transient frequency compared to Ctrl, as monitored with the single-wavelength fluorescent Ca^2 +^ indicator Fluo-4 (Fig. 5A, B; Videos S1 and S2). Treatment with the NMDAR antagonist NitroSynapsin normalized the aberrantly high intracellular Ca^2+^ levels and Ca^2+^ transient frequency in MHS hiPSC-neurons toward Ctrl values (Fig. 5C–E; Video S3). The concentration of drug used in these experiments was determined from our previously published dose-response curves that found 5–10 μM NitroSynapsin to be maximally efficacious and also attainable in vivo [30, 52, 69]. Notably, we had previously reported that NitroSynapsin ameliorates E/I imbalance in MHS-model mice, normalizing their aberrant synaptic activity, histological deficits, and ASD-like behavioral phenotypes [30].

Mechanistically, we and others have shown that compounds in the aminoadamantane class, such as memantine and the more efficacious NitroSynapsin, preferentially block extrasynaptic (e)NMDARs over synaptic receptors at the concentrations utilized here [50, 52, 69, 70]. Therefore, we next studied NMDAR-mediated currents in Ctrl and MHS hiPSC-neurons. We attempted to pharmacologically enrich for eNMDAR-associated responses using a previously published protocol [51, 52], although the synaptic vs. extrasynaptic origin of these responses may not be as definitively demarcated (for explanation, see legend to Fig. S8). Under these conditions, MHS hiPSC-neurons manifested an increase in basal NMDAR-mediated intracellular calcium responses compared to Ctrl (Fig. S8A, S8B), potentially due to an increase in basal extracellular glutamate levels in the MHS cultures [51, 52]. Critically, we found that NitroSynapsin could significantly decrease the NMDAR-mediated calcium responses in MHS cultures (Fig. S8A, 8B). Additionally, (2R)-amino-5-phosphonovalerate (APV), a well-known competitive NMDAR antagonist, also decreased these responses in MHS cultures (Fig. S8E, S8F). Moreover, treatment with the sodium channel blocker TTX abrogated the increase in basal NMDAR-mediated currents in MHS cultures and thus the effect of NitroSynapsin. These findings are consistent with the notion that excessive excitatory synaptic activity (as shown in Figs. 3 and 4) may cause glutamate spillover and consequently an increase in basal eNMDAR-mediated current (Fig. S8C, S8D), although a component of synaptic current contributing to this basal current cannot be excluded [30, 52, 71].

To further explore this effect of excessive NMDAR activity due to MEF2C haploinsufficiency on neural network activity, we employed multielectrode array (MEA) recordings in our Ctrl and MHS cultures. Similar to our measurements of Ca^2+^ transient activity, in MHS cultures compared to Ctrl we found an increase in mean firing rate, electrode burst frequency (reflecting primarily single neuron action potentials), network burst frequency (reflecting action potentials of multiple neurons), and synchronicity of firing (Fig. 5F–J). Notably, treatment with NitroSynapsin, to block excessive current (possibly eNMDAR-mediated), significantly decreased all of these parameters of aberrant hypersynchronous firing in MHS hiPSC-neuronal cultures while not affecting Ctrl cultures (Fig. 5F–J).

### Characterization of MHS vs. isogenic Ctrl in 3D cerebral organoids

**Fig. 6:**
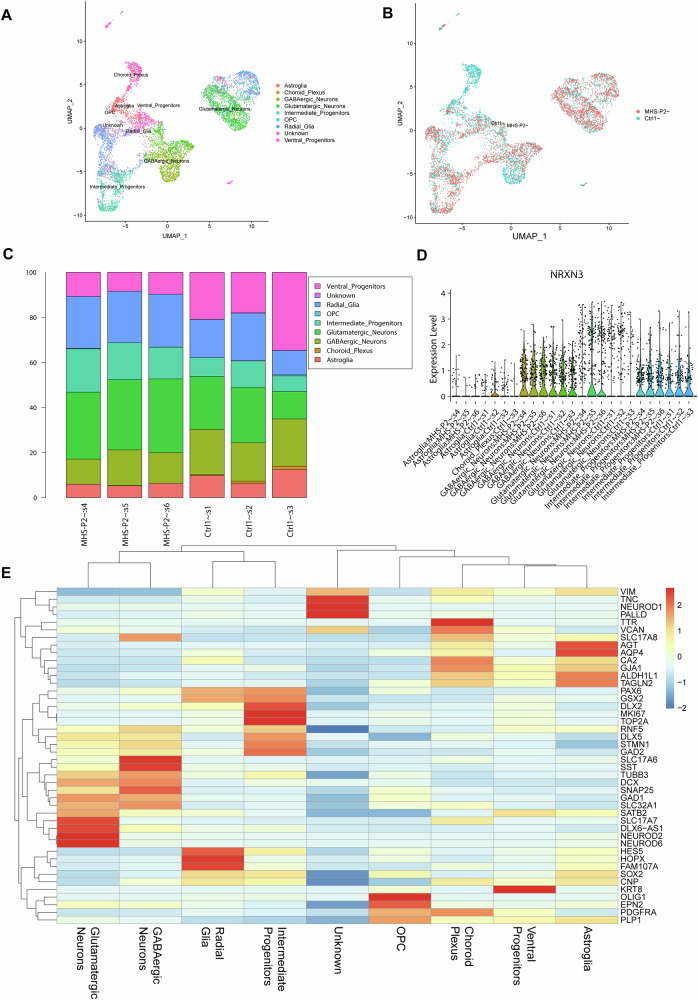
scRNA-seq analysis demonstrates reproducibility of Ctrl and MHS cerebral organoids at 3 months of age. Uniform Manifold Approximation and Projection (UMAP) analysis of isogenic Ctrl1 and MHS hiPSC-derived cerebral organoids by cell-type (**A**) and by genotype (**B**). **C** Bar charts showing relative cell-type composition of each individual organoid captured from scRNA-seq. **D** Violin plots showing the distribution of expression of NRXN3 by cell type. Note the increased expression of NRXN3 in MHS GABAergic neurons, in glutamatergic MHS neurons, and in progenitors. **E** Heatmap of key marker genes to annotated clusters.

To further validate our results in a more complex system than 2D cerebrocortical cultures, we next used hiPSC-derived cerebral organoids [34], a model that allows us to study neuronal circuits in 3D and better reflects in vivo microcircuits. To characterize the cellular content of our cerebral organoids and to rigorously establish the reliability and reproducibility of this model system [43, 45], we performed single cell RNA-sequencing (scRNA-seq) of stage-matched MHS and isogenic Ctrl cerebral organoids. By scRNA-seq, both MEF2C mutant and isogenic Ctrl reproducibly yielded similar cell types, including the presence of both excitatory/glutamatergic neurons (and their intermediate progenitors) and inhibitory/GABAergic neurons (and their ventral progenitors), in addition to other cell types (Fig. 6), with a statistically significant decrease in GABAergic neurons in the MHS organoids (*p* < 0.03) (Fig. 6A–C). Interestingly, we found an increased number of MEF2C transcripts within our GABAergic neuron population in the MHS compared to isogenic Ctrl organoid scRNA-seq data (Table S5). Given the decrease in MEF2 reporter gene activity we found in the MHS mutant population, this observation is consistent with the notion that our scRNA-seq may not be capturing full-length reads but rather abortive/non-functional partial transcripts due to mutation. This result may represent a compensatory feedback system at the promoter level. We also noted the presence of more RGCs, representing precursor cells for both astrocytes and glutamatergic neurons, in the MHS organoids over the Ctrl1 organoids (Fig. 6C). We carefully validated the expression of key markers across genotypes for each cell type to confirm their annotation (Fig. 6E).

Additionally, our scRNA-seq results on cerebral organoids showed increased NRXN3 in MHS patient GABAergic neurons (*p* < 0.006) compared to isogenic Ctrl (Fig. 6D and sheet 2 in Excel Table S4), corroborating our bulk RNA-seq data performed on 2D cultures. NRXN3 is important for synapse formation, differentiation, stabilization, and maturation [72]. Moreover, conditional knockout of *NRXN3* impairs AMPAR-mediated synaptic transmission and blocks NMDAR-mediated LTP in the hippocampus implying that increased NRXN3 could enhance excitability [73]. Recently it has been shown that NRXN3 in females suppresses GABA release from parvalbumin-positive neurons that synapse onto regular spiking neurons [74]. Thus, with many of our MHS patients being female, increased NRXN3 could contribute to the observed increase in excitability (Table S3). In support of this, one of our male patient samples (MHS-P1) displayed somewhat less hyperexcitability.

To further classify the cellular composition within our scRNA-seq data for the RGC and GABAergic clusters, we performed subclustering analysis on these populations (Fig. S9A, S9B). We then conducted a comprehensive analysis of various RGC and GABAergic interneuron markers to investigate potential differences between MHS and isogenic Ctrl WT organoids. The expression patterns of key genes associated with RGC and GABAergic neuron identity and function were examined (Fig. S9C). Our results revealed the presence of GABAergic-related transcripts in the subclusters derived from the GABAergic cluster, as evidenced for example by the expression of GAD1 and GAD2. Notably, we observed an increase in ARX, DLX1, DLX2, SLC32A1, GAD2, NPY, and CALB2 transcripts within subcluster 3 of GABAergic neurons in the MHS mutant organoids compared to isogenic Ctrl. This finding is consistent with an alteration in the development and specification of a subpopulation of GABAergic interneurons in the MHS cerebral organoids. We speculate that the heightened expression of these genes in MHS organoids may represent an abortive compensatory response to the MEF2C mutation, potentially aimed at preserving or reinstating GABAergic function through the upregulation of critical genes involved in interneuron development and functionality. Among RGCs subclusters, although we identified varying transcriptional signatures, no statistically significant differences were found in the profiles of these markers between MHS and isogenic Ctrl cerebral organoids.

Additionally, we performed a rank-rank hypergeometric overlap (RRHO) analysis to compare gene expression profiles from bulk RNA-seq and scRNA-seq data. Despite the different time points (5 weeks vs. 3 months) and culturing conditions for 2D vs. 3D cultures, the RRHO heatmap (Fig. S9D) reveals a significant region of overlap. The central red and yellow regions indicate strong concordance between the datasets, particularly for genes with mid-to-high expression levels. Peripheral blue regions show minimal overlap, suggesting less agreement for lower-ranked genes. These findings support the reliability of the single-cell data in reflecting the gene expression patterns observed in bulk RNA-seq, especially for genes with increased expression.

We also characterized our MHS and Ctrl cerebral organoids histologically at various time points to confirm the presence of neurons and astrocytes. As expected, the organoids formed rosettes and a stereotypical organization surrounding each rosette. We found that by 2-months in culture, MHS cerebral organoids compared to Ctrl were smaller in size (Fig. S10A, S10B), similar to human MHS patient brains in both children and adults [22, 24]. We also observed that MHS cerebral organoids recapitulated the 2D culture finding of a decreased proportion of neurons and neuropil compared to Ctrl, as judged by quantitative confocal immunofluorescence for β3-tubulin corrected for cell number (Fig. S10C–S10E). Moreover, we found a significant increase in GFAP expression by quantitative confocal immunostaining and by quantitative (q)PCR, but no difference in astrocyte-specific S100β staining (Fig. S10C and S10F–S10K). Since GFAP labels both RGCs/neural precursors and astrocytes, but S100β only astrocytes, this finding may possibly be attributed to the developmental stage of these organoids, at which time point neural progenitors were prominent (as confirmed by our scRNA-seq data), and gliogenesis was just beginning [75**–**77].

To investigate the developmental stage of the organoids further, we performed additional immunocytochemical studies to better understand the cellular composition and differentiation (Fig. S11). MHS cerebral organoids showed an increase in nestin-positive hNPCs, indicating an abnormal build-up of early neural progenitors compared to Ctrl (Fig. S11A, S11B). We also found increased expression of BLBP, found in both RGCs and astrocytes [78], in the MHS organoids compared to isogenic Ctrl (Fig. S10F, S10G). This observation is consistent with the elevated nestin expression in these samples, suggesting an expanded population of hNPCs (Fig. S10F, S10G), some of which differentiate into astrocytes. On the other hand, we found a decrease in TBR2 labeling in MHS cerebral organoids, consistent with a delay or stall in intermediate progenitor cell development compared to Ctrl at this stage (Fig. S11A, S11C).

There was also evidence in the immunocytochemical staining for disruption of cortical layer formation in the MHS compared to Ctrl organoids, similar to that reported for conditional knockout of *MEF2C* in mouse brain [11**–**14]. To this point, CTIP2, a cerebrocortical layer V-VI marker, and TBR1, a cortical layer VI marker, were markedly decreased in expression in MHS cerebral organoids compared to Ctrl (Fig. S11A, S11D and S11E). Additionally, immunocytochemistry confirmed that the number of cells expressing GABA was increased in Ctrl compared to MHS organoids (Fig. S11H, S11I), in agreement with the scRNA-seq data (Fig. 6A–C). Overall, these immunocytochemical findings provide added context to our transcriptomic data, highlighting posttranscriptional changes not necessarily captured in the RNA sequencing analysis.

### NitroSynapsin corrects hypersynchronous excessive activity in MHS cerebral organoids

**Fig. 7:**
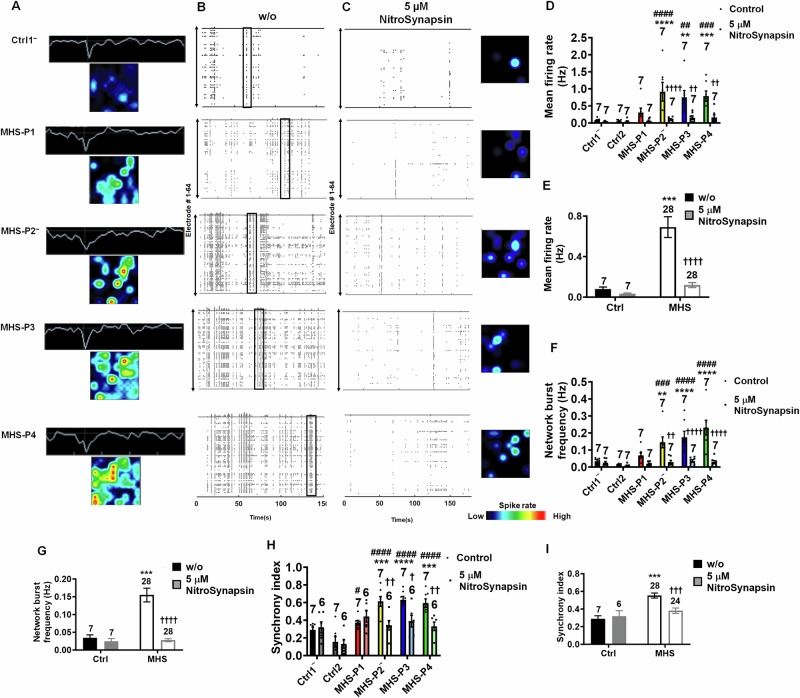
NitroSynapsin abrogates hypersynchronous burst activity in MHS cerebral organoids. **A** Representative heat maps and single traces from Ctrl1 and MHS hiPSC-derived cerebral organoids in individual MEA wells at 3–4 months of age. **B** Representative raster plots of MEA recordings in Ctrl and MHS cerebral organoids. Boxes outline examples of network bursts. **C** Representative raster plots and heat maps of MEA recordings in Ctrl and MHS cerebral organoids after treatment with NitroSynapsin (NitroSyn). **D**–**I** Quantification of MEA mean firing rate, network burst frequency, and synchrony index. Each MHS patient’s cerebral organoids vs. each control shown in **D**, **F** and **H**; or grouped MHS patient cerebral organoids vs. controls shown in **E**, **G** and **I**. Data are mean ± SEM. Sample size is listed above bars from 6 to 7 separate cerebral organoids recorded for each genotype. *^,#,†^ *p* < 0.05, **^,##,††^*p* < 0.01, ***^,###, †††^*p* < 0.001, ****,^####,††††^*p* < 0.0001 by ANOVA with Sidak’s post-hoc test for comparison to Ctrl1 (*) or to Ctrl2 (^#^), or within a group (^†^) between NitroSynapsin treatment vs. without (w/o) treatment (see Methods).

In MEA recordings, we found a dramatic increase in electrical activity in MHS cerebral organoids compared to Ctrl (Fig. 7A, B and D). Similar to our 2D cultures described above, MHS organoids showed increased frequency of network bursts and firing synchrony compared to Ctrl (Fig. 7E, F). Importantly, NitroSynapsin normalized the excessive mean firing rate, network burst frequency, and firing synchrony of the MHS cerebral organoids virtually to Ctrl levels, while having minimal effect on Ctrl organoids (Fig. 7C–F). Additionally, we found that APV inhibited neuronal activity in cerebral organoids across all genotypes, suggesting that the bursting activity observed was indeed NMDAR-mediated (Fig. S12A, S12B). Quantification of percent inhibition of mean firing rate of control cerebral organoids by APV and NitroSynapsin showed that the effect of NitroSynapsin was significantly less than that of APV. In contrast, NitroSynapsin more robustly inhibited the abnormal activity observed in MHS organoids than APV (Fig. S12C). These findings are consistent with the results reported here as well as previously published findings that NitroSynapsin preferentially inhibits excessive activity of NMDARs while relatively sparing normal/physiological receptor activity [50, 52, 69, 70].

## Discussion

As demonstrated in murine models, MEF2C plays a critical role during development to shape neuronal connectivity [14, 30, 79]. At early developmental stages, MEF2C is important for transcription of neuronally restricted genes and for the differentiation of hNPCs into neurons [11]. In addition, MEF2C regulates formation and spread of dendrites, contributing to dendritic complexity and synapse formation, ensuring structural integrity of the neural network [11, 14, 30, 80]. In contrast, at slightly later stages of development onward, MEF2C plays an important role in excitatory synapse pruning, promoting the formation and maintenance of the adult neural network [66, 81]. Interestingly, heterozygosity for *MEF2C* in mice results in increased electrical excitability [30]. Here, we found similar results in 2D cultures and cerebral organoids in the context of the human MHS form of ASD/ID using patient-derived hiPSCs bearing *MEF2C* mutations studied after up to 3–4 months in culture.

These developmental aspects of the effect of MEF2C may account for several of our findings. For example, although we observed a decrease in synapsin-I/PSD-95 punctae in Fig. S7, we also found increased VGLUT1 intensity, as shown in Fig. 4, which could be the reason for increased mEPSC frequency. The observed effect could occur because of an imbalance between pruning of synapses and development of new synapses, as in both cases MEF2C is known to be involved. MEF2C deficiency prevents the brain from removing unwanted excitatory synapses, which would otherwise be eliminated [14, 66–68]. On the other hand, in initial stages of development, MEF2C plays a significant role in the formation of new synapses [11, 30, 80]. Accordingly, in Fig. 4, we document a decrease in VGAT level in MHS neurons, consistent with a decrease in inhibitory presynaptic structures and abnormal GABAergic neuron development (as shown previously in MHS mice) [30, 65], thus contributing to E/I imbalance. These findings are also reflected in the increased excitability that we observed in network recordings from MHS hiPSC-neurons, in some cases resembling MHS patient seizures (Video S2), with the exception of MHS-P1. The lack of significantly increased excitation in MHS-P1 may reflect the lack of seizures in this patient compared to the others (Table S1).

**Fig. 8:**
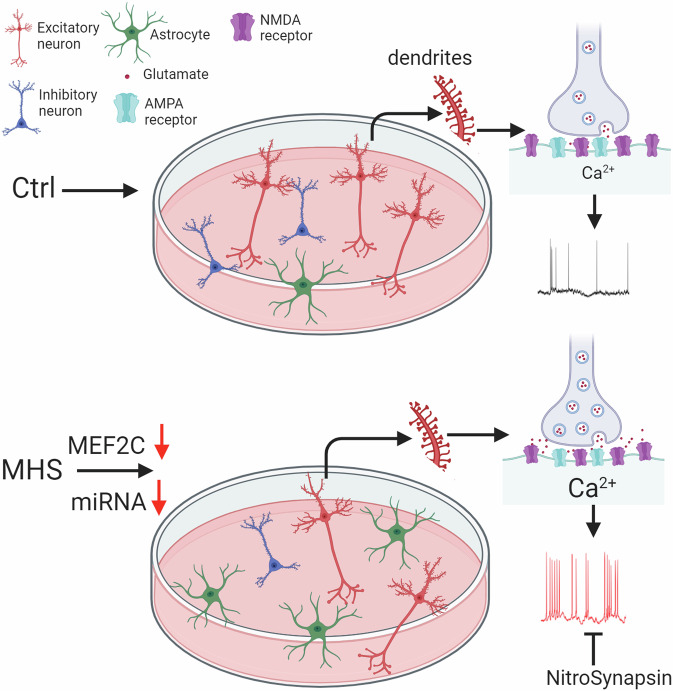
Aberrant neurogenesis/gliogenesis and excitation in MEF2C autism patient hiPSC-neurons. Schematic diagram showing that MHS**-**hiPSCs generate more astrocytes and fewer cerebrocortical neurons. Compared to control (Ctrl), the MHS neuronal population consists of fewer inhibitory neurons and excitatory neurons, but with more VGLUT1 vesicles, resulting in increased presynaptic glutamate release, increased postsynaptic intracellular Ca^2+^ levels, and hence increased excitability. The novel NMDAR antagonist NitroSynapsin ameliorates this hyperactivity.

Accounting for the increase in excitability, MHS hiPSC-neurons displayed an increase in NMDAR activity, possibly mediated at least in part by eNMDARs, consistent with overflow of neurotransmitter from hyperactive excitatory synapses (schematically summarized in Fig. 8). We also show that very early in development (e.g., 2D cultures at 1 month) human MEF2C haploinsufficiency yields hiPSC-derived cultures with more astrocytes but fewer neurons, at least in part due to aberrantly low miRNA (miR-4273 and miR-663) expression. Mechanistically, miR-663 has been reported to affect genes involved in neurogenesis vs. gliogenesis [54, 55]. Additionally, this increase in astrocytes, as observed in our GFAP and S100β staining in both 2D MHS cultures and 3D MHS cerebral organoids, could also have modulated neuronal electrical activity in MHS patient neurons [82, 83].

In mice, MEF2C has been shown to regulate genes involved in inhibitory synapse formation [67] and is essential for the development of PV-expressing inhibitory neurons [65]. In MHS hiPSC-neurons, we found decreased expression of the inhibitory vesicular protein VGAT accompanied by a decrease in mIPSC frequency, revealing dysfunctional inhibitory synapse activity. Along similar lines, systemic MEF2C haploinsufficiency in mice (with lower MEF2 activity as seen in human MHS patients) resulted in decreased mIPSC frequency [30]. Seemingly, in contrast to these findings, conditional knockout of *MEF2C* in embryonic cortical excitatory neurons (using *Emx1*^*Cre/+*^ knock-in mice crossed with *MEF2C*^*fl/fl*^ mice) led to a moderate decrease in excitatory transmission and a large increase in inhibitory transmission [16]. However, those findings were the result of excitatory neuron-specific deletion of *MEF2C*, while inhibitory neurons were spared since cortical excitatory neurons and glia but not GABAergic neurons are produced in the Emx1-expressing lineage [84]. Likewise, MEF2C-haploinsufficient hiPSCs, converted to excitatory neurons using lentiviral overexpression of neurogenin2, exhibited decreased excitation compared to controls [85]. Notably, a similar decrease in excitatory synaptic activity was observed with conditional brain knockout of *MEF2C* in *NESTIN-Cre*^*+*^*/MEF2C*^fl/Δ2^ conditional null mice (where Δ2 represents exon 2-deleted allele) [11], arguing that this phenotype is due to complete loss of MEF2C activity at a very early stage of development.

As found in the present study in MHS hiPSC-derived human neurons, where approximately half of MEF2C activity is lost, the increase in excitatory synaptic and extrasynaptic activity as well as the decrease in inhibitory synaptic transmission disrupts the excitatory-inhibitory (E/I) ratio. This contributes to hypersynchronous burst activity in neural networks. Several studies have associated E/I imbalance with ASD [30, 86–88]. As recently reported for *MEF2C* heterozygous mice [30], we found that the NMDAR antagonist, NitroSynapsin, which preferentially inhibits excessive/pathological NMDAR activity while relatively sparing physiological activity [50, 52, 69, 70], abrogated the aberrant network hyperactivity in MHS hiPSC-derived neuronal cultures and cerebral organoids. This report is the first hiPSC-based study to test NitroSynapsin previously used in the ASD animal model of MHS. These findings suggest that this drug merits further study for its therapeutic potential. Moreover, since MEF2C has been shown to regulate expression of many hub genes known to be associated with ASD/ID [20, 21, 59], and excessive levels of glutamate may contribute to these phenotypes [89], a similar approach may prove effective in for other forms of ASD/ID.

### Limitations of the study

One limitation is the limited number of hiPSC lines evaluated here. This is largely offset, however, by the fact that an isogenic control was also employed, greatly increasing the power of the findings [90]. Another potential limitation concerns lack of definitive parcellation of cell types by scRNA-seq, immunocytochemistry, electrophysiology, or other omic methods, especially during development of the brain when phenotypes are changing and in neurodevelopmental diseases where intermediate phenotypes might be observed. Nonetheless, the platforms presented here provide a model system based on hiPSC-derived 2D cultures and 3D cerebral organoids on which to begin study of neurodevelopmental diseases by comparing mutant to carefully selected controls. Of note, MEF2C is not expressed in astrocytes, and therefore, we concentrated on effects on neurons here. On the other hand, MEF2C is also expressed in brain microglia, and this is being explored in a separate set of experiments. We also acknowledge that our findings of decreased neurogenesis and increased gliogenesis (of astrocytes) very early on in development may not be directly linked, and their association will require further study. Moreover, despite our use of hiPSCs from both sexes, potential biases could include lack of adequate representation of different races, geographic regions (where environmental factors could come into play), and patient fibroblast samples obtained at various ages, to name just a few. In spite of these limitations, this is the first study to faithfully reproduce many of the features of human MHS in hiPSC-based model systems, including the lack of normal neuronal and synaptic differentiation, and the presence of a hyperelectrical phenotype, as observed on EEGs of children with the MHS form of ASD/ID.

## Supplementary information

supplementary information

Supplementary information file 1

Table S2

Table S4

Table S5

Video S1

Video S2

Video S3

## Data Availability

• Original data for ChIP-Seq and RNA-Seq will be available online. • The data sets generated during the current study are available within the paper or from the corresponding author on reasonable request. • This paper does not report original code. • Any additional information required to reanalyze the data reported in this paper is available from the Lead Contact upon request.

## Materials availability

All biological resources, antibodies, cell lines and model organisms and tools are either available through commercial sources or the corresponding authors. All unique/stable reagents generated in this study are available from the Lead Contact with a completed standard institutional Materials Transfer Agreement (MTA). Further information and requests for resources and reagents listed in Key Resources Table should be directed to the Lead Contact.
