# Supplementary material for: Dysregulation of miRNA expression and excitation in MEF2C autism patient hiPSC-neurons and cerebral organoids
Source: Mol Psychiatry. 2024 Sep 30;30(4):1479–96. doi: 10.1038/s41380-024-02761-9 (PMC11919750; doi:10.1038/s41380-024-02761-9)
Supplement: Supplementary file 1 — supplementary information [file 41380_2024_2761_MOESM1_ESM.docx]

**Supplementary Information**

Dysregulation of miR expression and excitation in MEF2C autism patient hiPSC-neurons and cerebral organoids

Dorit Trudler, Swagata Ghatak, Michael Bula, James Parker, Maria Talantova, Melissa Luevanos, Sergio Labra, Titas Grabauskas, Sarah Moore Noveral, Mayu Teranaka, Emily Schahrer, Nima Dolatabadi, Clare Bakker, Kevin Lopez, Abdullah Sultan, Parth Patel, Agnes Chan, Yongwook Choi, Riki Kawaguchi, Pawel Stankiewicz, Ivan Garcia-Bassets, Piotr Kozbial, Michael G. Rosenfeld, Nobuki Nakanishi, Daniel H. Geschwind, Shing Fai Chan, Wei Lin, Nicholas J. Schork, Rajesh Ambasudhan, and Stuart A. Lipton

**Supplementary Figures and Figure Legends**

**
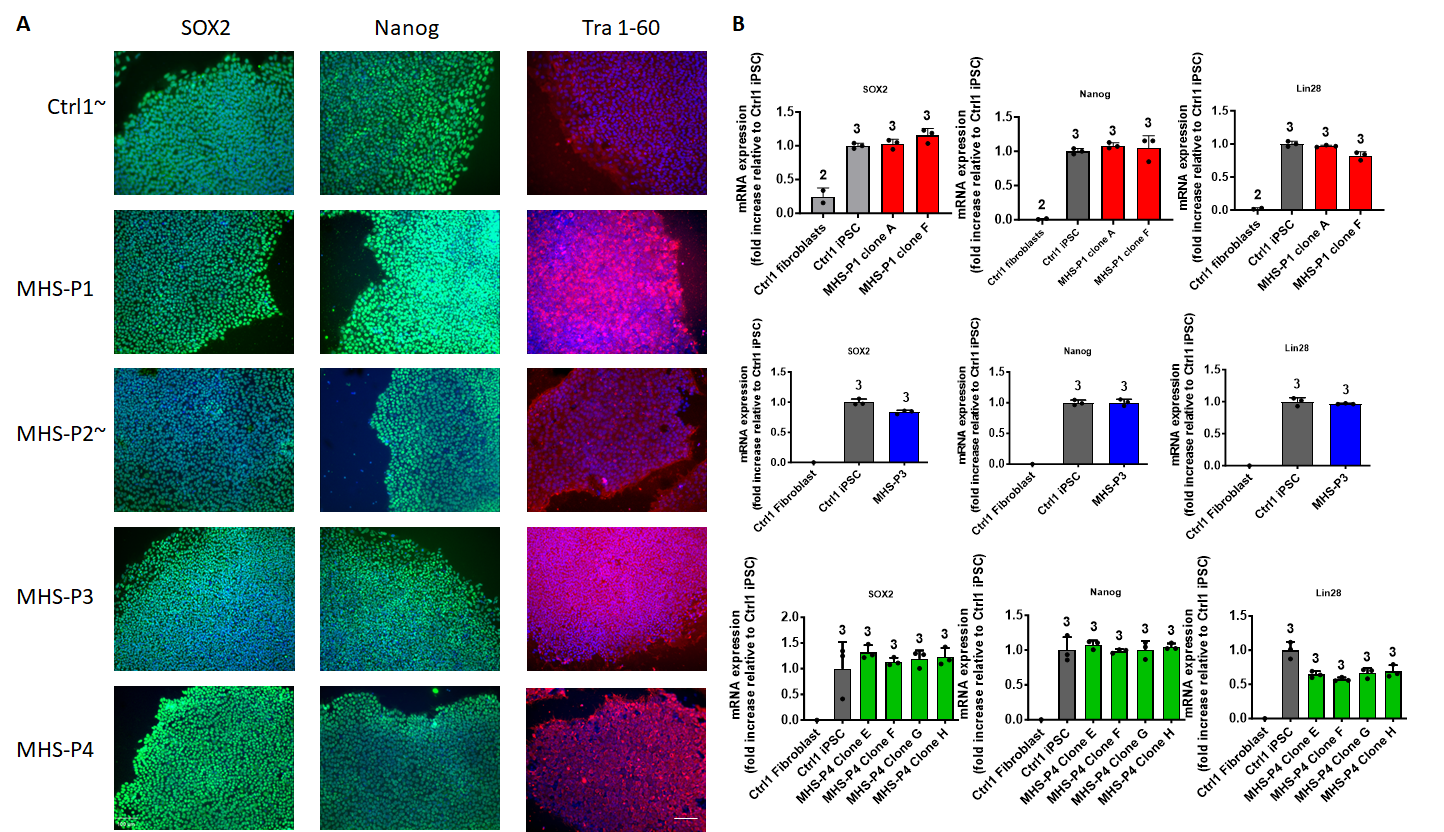
**

**Fig. S1 Generation of hiPSCs in 2D cultures from patient fibroblasts.**

**(A)** Immunocytochemistry showing expression of pluripotency markers SOX2, Nanog and Tra1-60 in all hiPSC lines. Scale bar, 100 µm.

**(B)** qPCR analysis of pluripotency markers SOX2, Nanog and Lin28 for several clones generated for each MHS patient hiPSC vs. Ctrl fibroblasts. Data are mean + SEM. Sample size listed above bars (number of replicates).


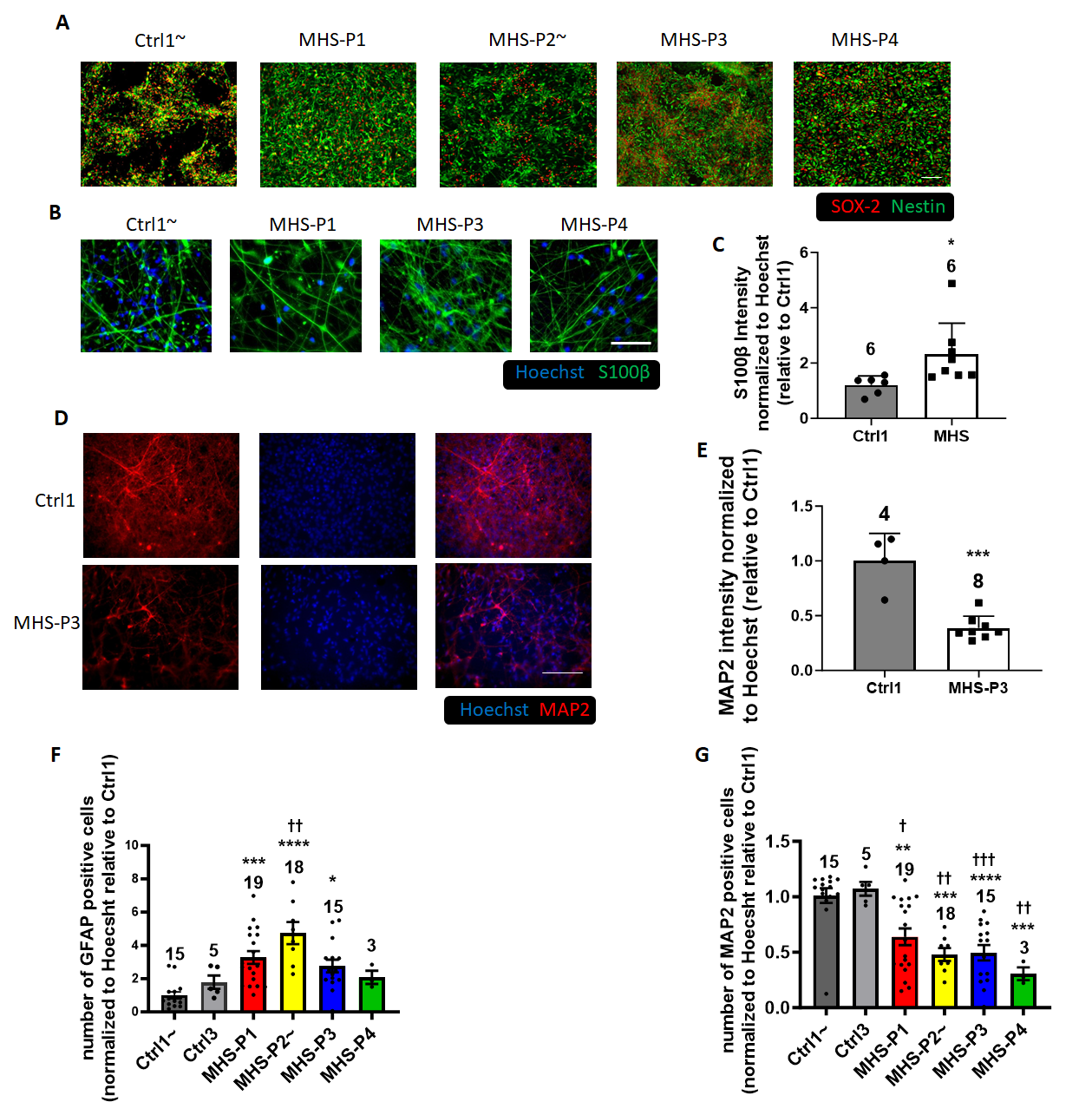


**Fig. S2 MHS patient hNPC characterization in 2D cultures.**

**(A)** Immunocytochemistry of SOX2 and nestin markers in Ctrl and MHS patient hiPSC-derived hNPCs. Scale bars, 100 µm.

**(B)** Immunocytochemistry of S100β and Hoechst in neurons differentiated from Ctrl and MHS hiPSCs after 1 month. Scale bars, 50 µm.

**(C)** Quantification of S100β expression.

**(D)** Immunocytochemistry of MAP2 in Ctrl1 and MHS-P3 patient hiPSC-derived neurons at 3 months. Scale bars, 100 µm.

**(E)** Quantification of MAP2 expression in 3-month-old neurons from Ctrl and MHS.

(F) Quantification of number of GFAP positive cells for each MHS line normalized to Hoechst and relative to Ctrls from 1-month-old hiPSC-derived cultures.

(G) Quantification of number of MAP2 positive cells for each MHS line normalized to Hoechst and relative to Ctrls 1-month old hiPSC-derived cultures.

Data are mean + SEM. Sample size listed above bars (number of replicates in 3 independent experiments). **p* < 0.05, ****p* < 0.001 by Student’s t test for C and E. **p* < 0.05, ***p* <0.01, ****p* < 0.001, *****p* < 0.0001 by ANOVA with Sidak’s post hoc test for multiple comparisons.

**
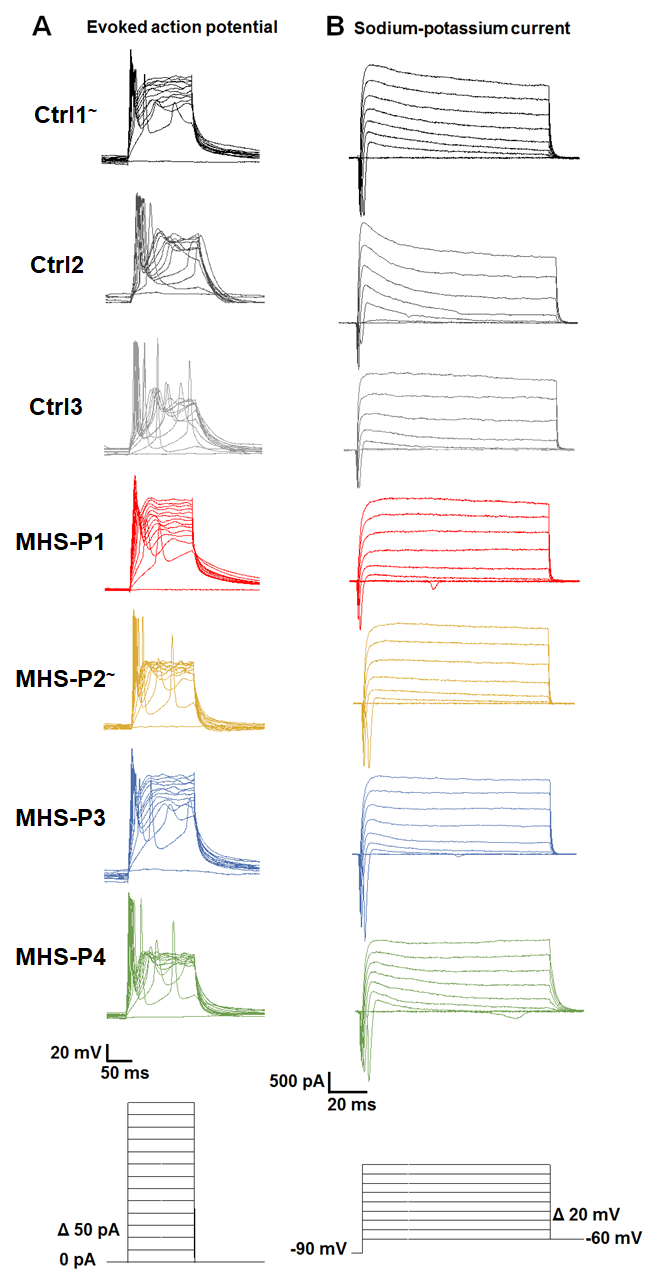
**

**Fig. S3 Both Ctrl and MHS hiPSC-derived cerebrocortical neurons in 2D cultures fire action potentials (APs) in current clamp, and manifest sodium and potassium currents under voltage-clamp.**

**(A)** Patch-clamp recording of evoked APs in hiPSC-derived neurons from a holding potential (V_h_) of -60 mV in current-clamp mode. Current-injection protocol used to evoke APs illustrated below traces.

**(B)** Representative sodium and potassium currents in voltage-clamp mode elicited by voltage steps from V_h_ = -60 mV after a prepulse to -90 mV for 300 ms. Voltage protocol illustrated below traces.


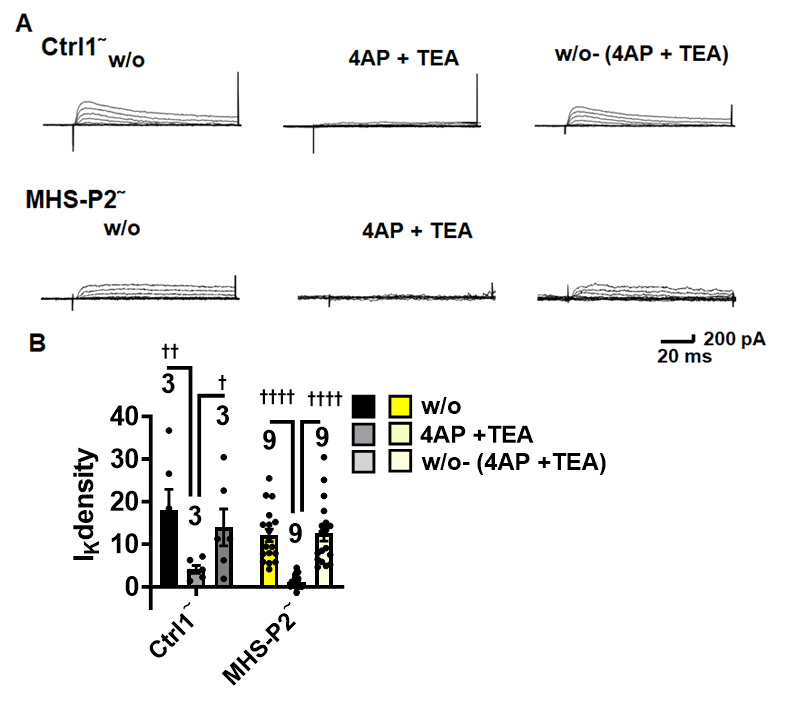


**Fig. S4** **Whole-cell currents of Ctrl and MHS hiPSC-derived cerebrocortical neurons in 2D cultures affected by 4AP and TEA.**

**(A)** Representative traces of outward currents. Currents were elicited in MHS-P2 and isogenic control neurons before (w/o) and after treatment with 5 mM 4AP and 20 mM TEA from a holding potential of -70 mV by voltage steps in 10 mV increments from -70 to +30 mV after a prepulse to -90 mV for 300 ms in the presence of 1µM TTX to inhibit sodium current. Subtracted traces (baseline currents minus drug-treated currents, indicated by w/o (4AP + TEA)) shown in *right-hand panel*.

**(B)** Quantification of 4AP/TEA-sensitive (potassium, I_K_) current density. Our analysis considered both peak and steady-state I_K_ values. Number of neurons recorded listed above bars from 3 different experimental cultures. Data are mean ± SEM. ^†^*p* <0.05, ^††^*p* <0.01, ^†††^*p* < 0.001, ^††††^*p* < 0.0001 by ANOVA with Sidak’s post-hoc test for comparison within a group (^†^) comparing “without treatment” (w/o) or subtracted traces vs. after 4AP + TEA treatment.


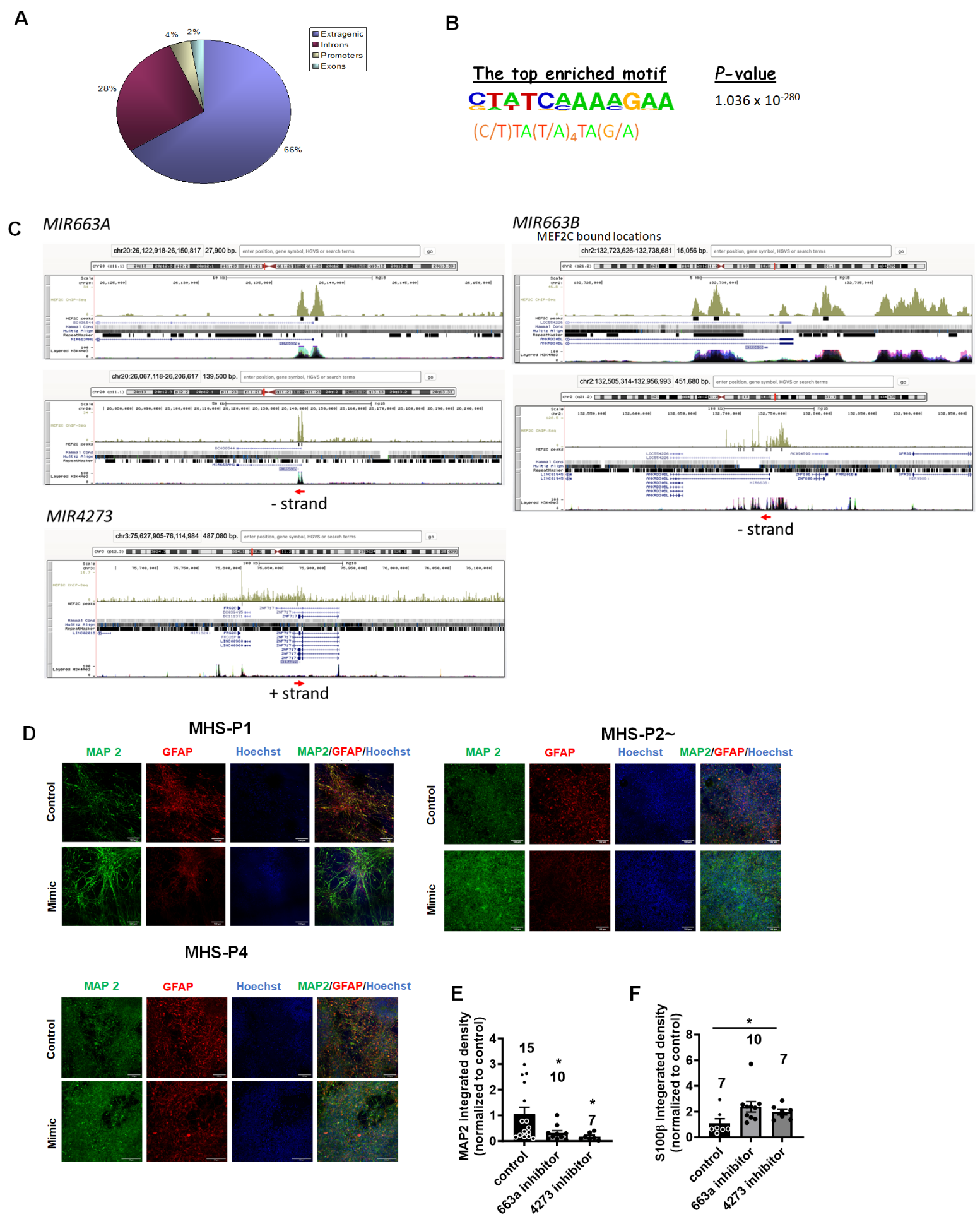


**Fig. S5 ChIP-seq for MEF2 targets and miRNA mimic.**

**(A)** Pie chart showing the distribution of MEF2 binding sites on the genome. Blue indicates extragenic sequences that bind MEF2.

**(B)** Top-enriched motif for MEF2 binding.

**(C)** ChIP-seq datasets for miR663a, miR663b and miR4273 in the vicinity of the *MEF2C* gene locus.

**(D)** Representative images of GFAP, MAP2, Hoechst (to label cell nuclei), and merged images in hiPSC-derived 2D cultures following exposure to miRNA4273 mimic or control. Scale bar, 100 µm.

**(E)** MAP2 neuronal marker expression in Ctrl1 hiPSC-derived cells expressing miRNA inhibitors compared to non-target control inhibitor after 2 weeks in culture.

**(F)** S100β astrocytic marker expression in Ctrl1 cells expressing miRNA inhibitors compared with non-target control inhibitor after 2 weeks in 2D cultures. Data are mean + SEM. Sample sizes (n) are listed above bars from at least 3 independent experiments. **p* < 0.05, by ANOVA with Dunnett’s post-hoc test for multiple comparisons or by two-tailed Student’s t test for single comparisons.

**
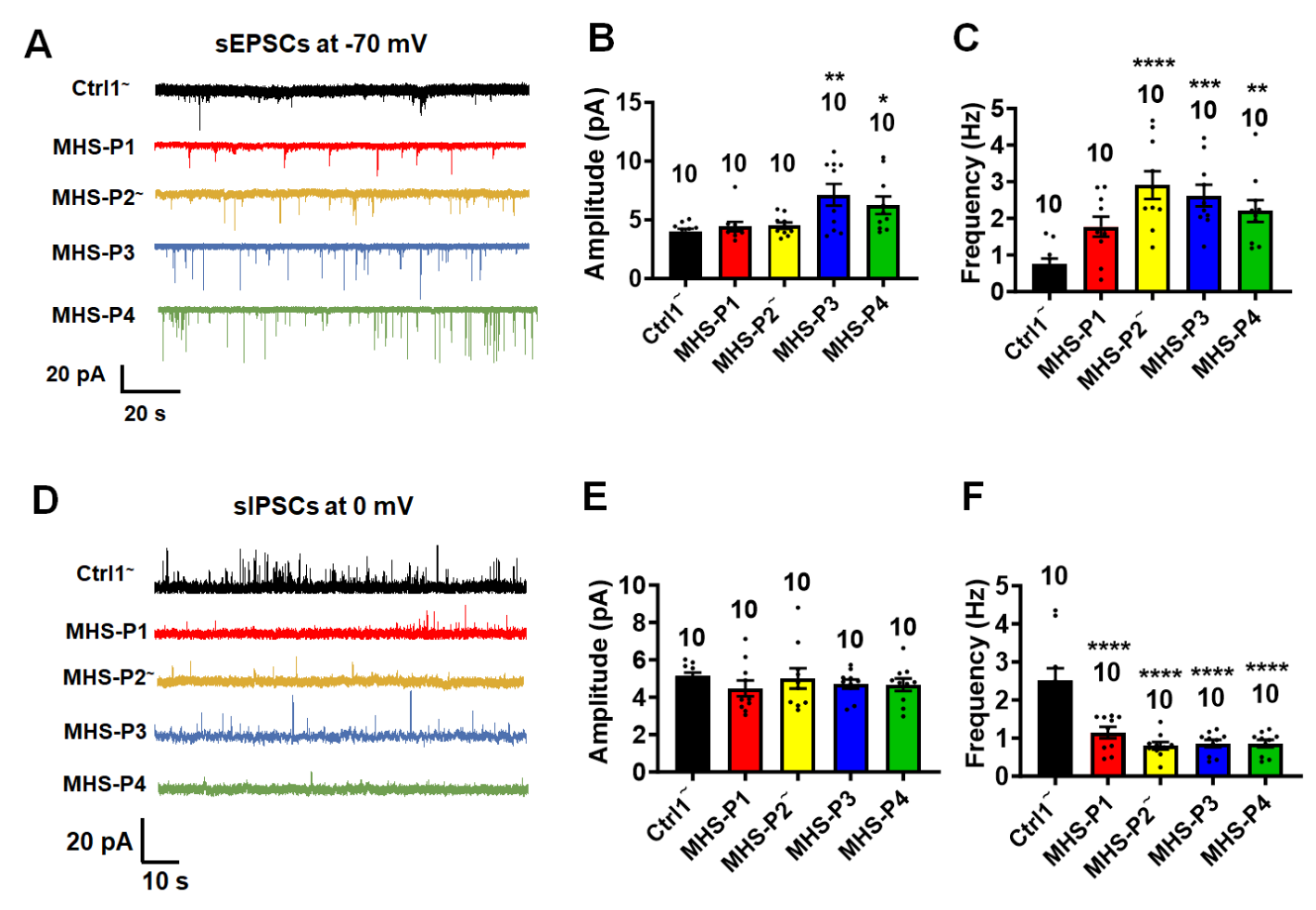
**

**Fig. S6 MHS hiPSC-derived cerebrocortical neurons in 2D cultures exhibit increased excitatory synaptic activity and decreased inhibitory synaptic activity.**

**(A)** Representative spontaneous (s)EPSCs recorded at -70 mV from Ctrl and MHS hiPSC-neurons in culture for 5 weeks.

**(B** and **C)** Quantification of sEPSC mean amplitude and frequency.

**(D)** Representative sIPSCs recorded at 0 mV from Ctrl and MHS hiPSC-neurons in culture for 5 weeks. **(E** and **F)** Quantification of sIPSC mean amplitude and frequency. Data are mean ± SEM. Sample size (n) listed above bars from 3-5 experiments. **p* < 0.05, ***p* < 0.01, ****p* <0.001, *****p* < 0.0001 by ANOVA with Dunnett’s post hoc test for multiple comparisons with Ctrl1.

**
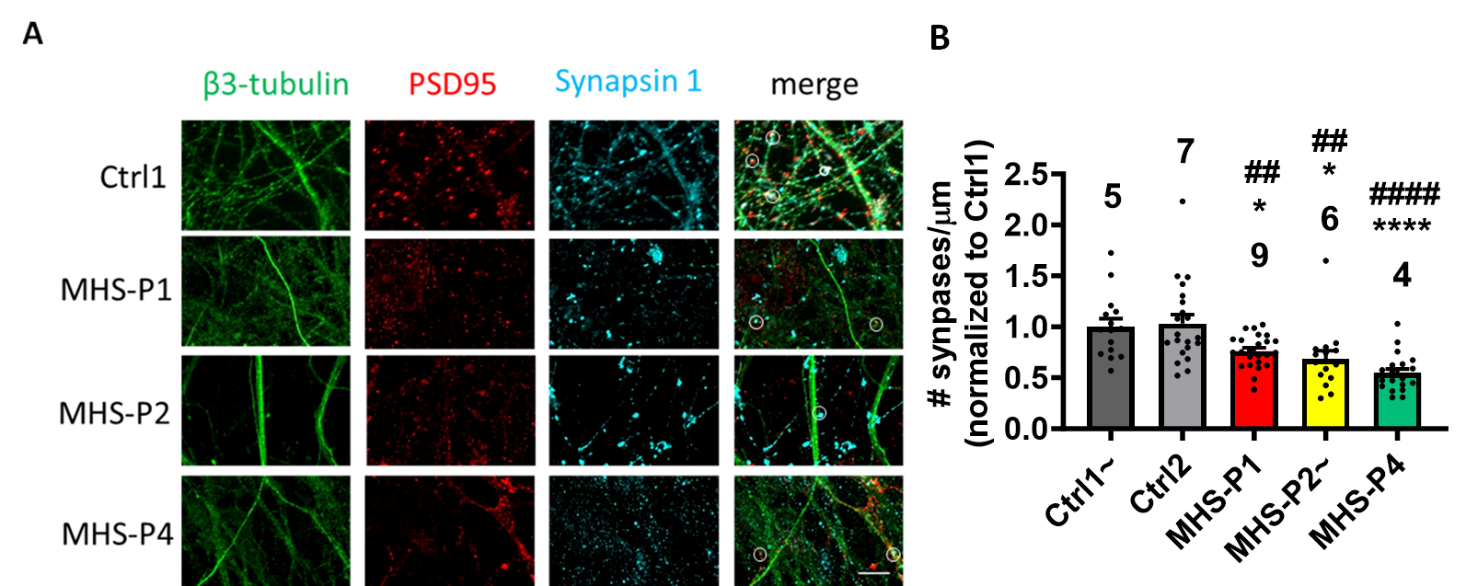
**

**Fig. S7** **MHS hiPSC-derived cerebrocortical neurons in 2D cultures exhibit fewer synapses.**

**(A)** Representative images of β3-tubulin, PSD-95, synapsin I, and merged image for Ctrl and MHS patients. Scale bar, 10 µm.

**(B)** Quantification of number of synapses (coincident synapsin I/PSD-95 punctae staining, marked by white circles) per neurite length for Ctrl and MHS patients. Data are mean + SEM. Number of imaged fields (n) listed above bars from 3 separate experiments. **p* < 0.05, *****p* < 0.0001 by ANOVA with Dunnett’s post hoc test for multiple comparisons.

**
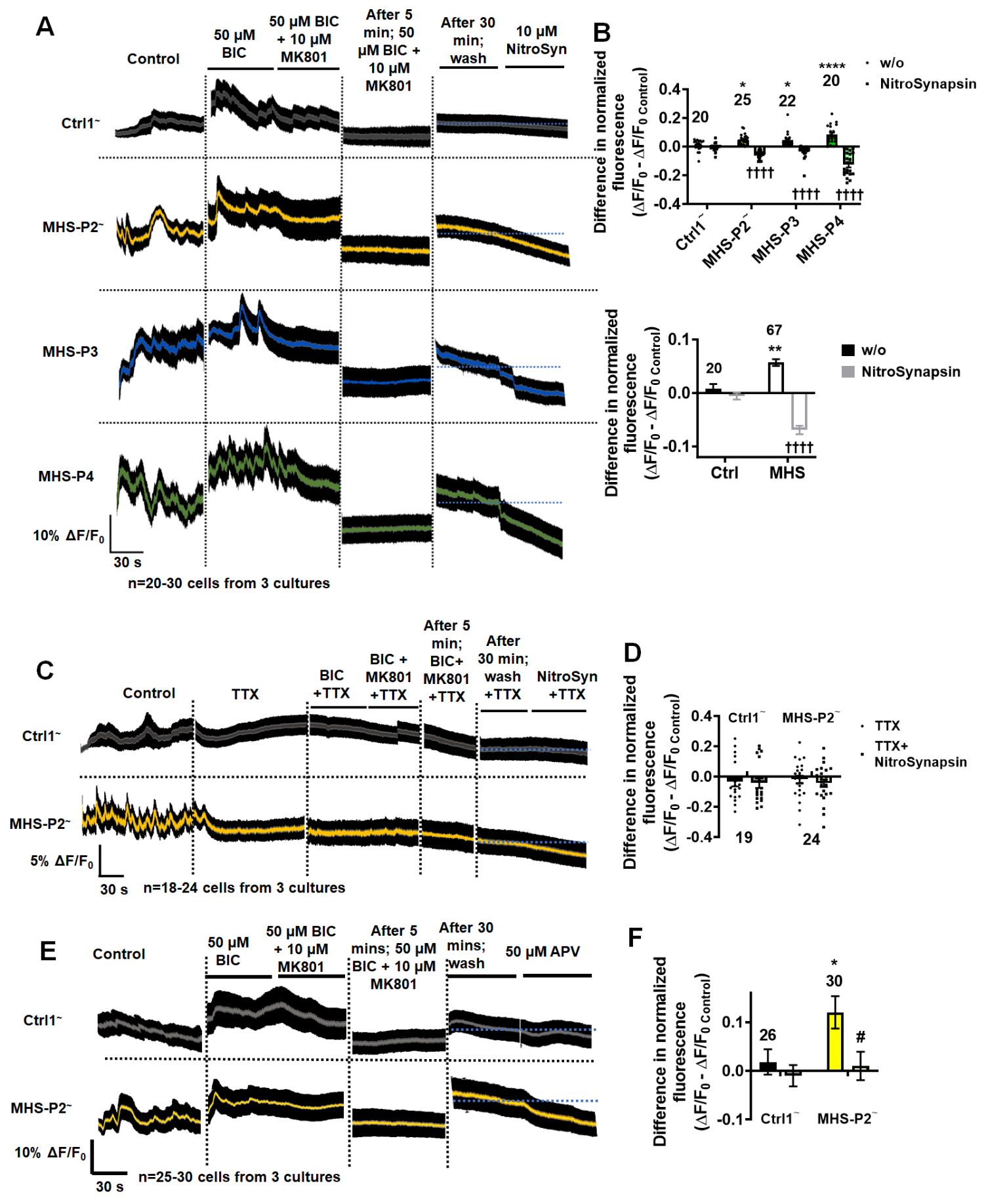
**

**Fig. S8 NitroSynapsin inhibits aberrant basal NMDAR-responses in MHS hiPSC-derived cerebrocortical neurons in 2D cultures.**

**(A)** Mean ± SEM envelope of calcium responses and their inhibition by NitroSynapsin (NitroSyn) monitored with Fluo-4 in Ctrl and MHS neurons after pharmacological enrichment of putative eNMDARs. The protocol for studying eNMDAR responses consisted of activation and subsequent blockade of synaptic NMDAR-mediated currents by bicuculline (BIC) and MK-801, respectively. Note, however, that the efficacy of this protocol is predicated upon sufficient numbers of inhibitory synaptic responses being antagonized by BIC in order to activate excitatory synaptic transmission; this may not have occurred in all cultures, as MEF2C haploinsufficiency has been shown to decrease inhibitory synapse formation. Hence, an additional synaptic component of the responses studied here cannot be ruled out. Blue-dotted line added to show Ca^2+^ response before and after NitroSynapsin.

**(B)** Quantification of basal NMDAR-mediated Ca^2+^ responses and effect of NitroSynapsin. Responses before treatment (bright bars with positive values, signifying the amplitude of the presumptive eNMDAR-mediated response) and after treatment (NitroSynapsin; pale bars with negative values, representing inhibition of the NMDAR-mediated response) in Ctrl and MHS neurons.

**(C)** Mean ± SEM envelope of calcium traces from Ctrl and MHS hiPSC-neurons after pharmacological enrichment for eNMDAR-mediated responses in the presence of TTX.

**(D)** Quantification of NMDAR-mediated Ca^2+^ responses in the presence of TTX.

(**E**) Mean ± SEM envelope of calcium traces showing inhibition with APV in Ctrl and MHS neurons of NMDAR-mediated responses.

(**F**) Quantification of NMDAR-mediated Ca^2+^ responses in the presence of APV *^,#^*p* < 0.05, ****^,####^*p* < 0.0001 by ANOVA with post hoc Dunnett’s test for comparison to Ctrl (*) or with post hoc Sidak’s test between w/o and with NitroSynapsin (^#^) for each genotype or with unpaired t-test for single comparisons.


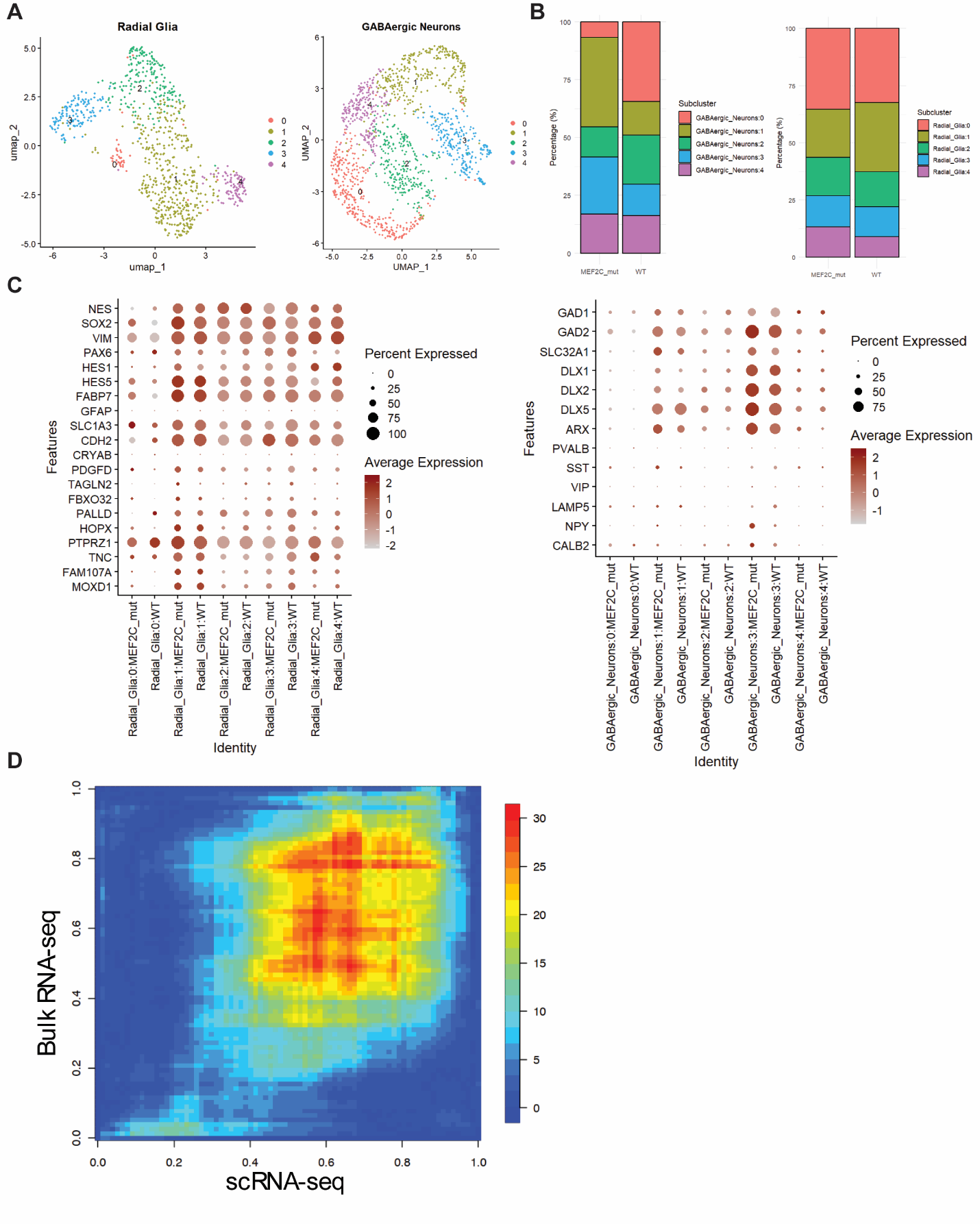


**Fig. S9 Integrated analysis of RGC and GABAergic populations in MHS and control cerebral organoids with comparison to 2D cultures.**

**(A)** Refined UMAP visualization of RGC (left) and GABAergic (right) subclusters in isogenic Ctrl1 and MHS hiPSC-derived cerebral organoids.

**(B)** Bar charts showing relative cell-type composition of each subcluster of RGCs (left) and GABAergic neurons (right) from the scRNA-seq data.

**(C)** Dot plots showing expression of key RGC and GABA interneuron markers across subclustered populations from the scRNA-seq data.

**(D)** Concordance in differential expression based on a rank-rank hypergeometric overlap (RRHO) plot comparing gene expression patterns between 3-month organoid-derived scRNA-seq data (x-axis) and 5-week hiPSC-derived neuron bulk RNA-seq data (y-axis). Heatmap colors represent the -log(P-values), with larger values indicating higher concordance. The top-right corner signifies genes that are upregulated in both datasets.

**
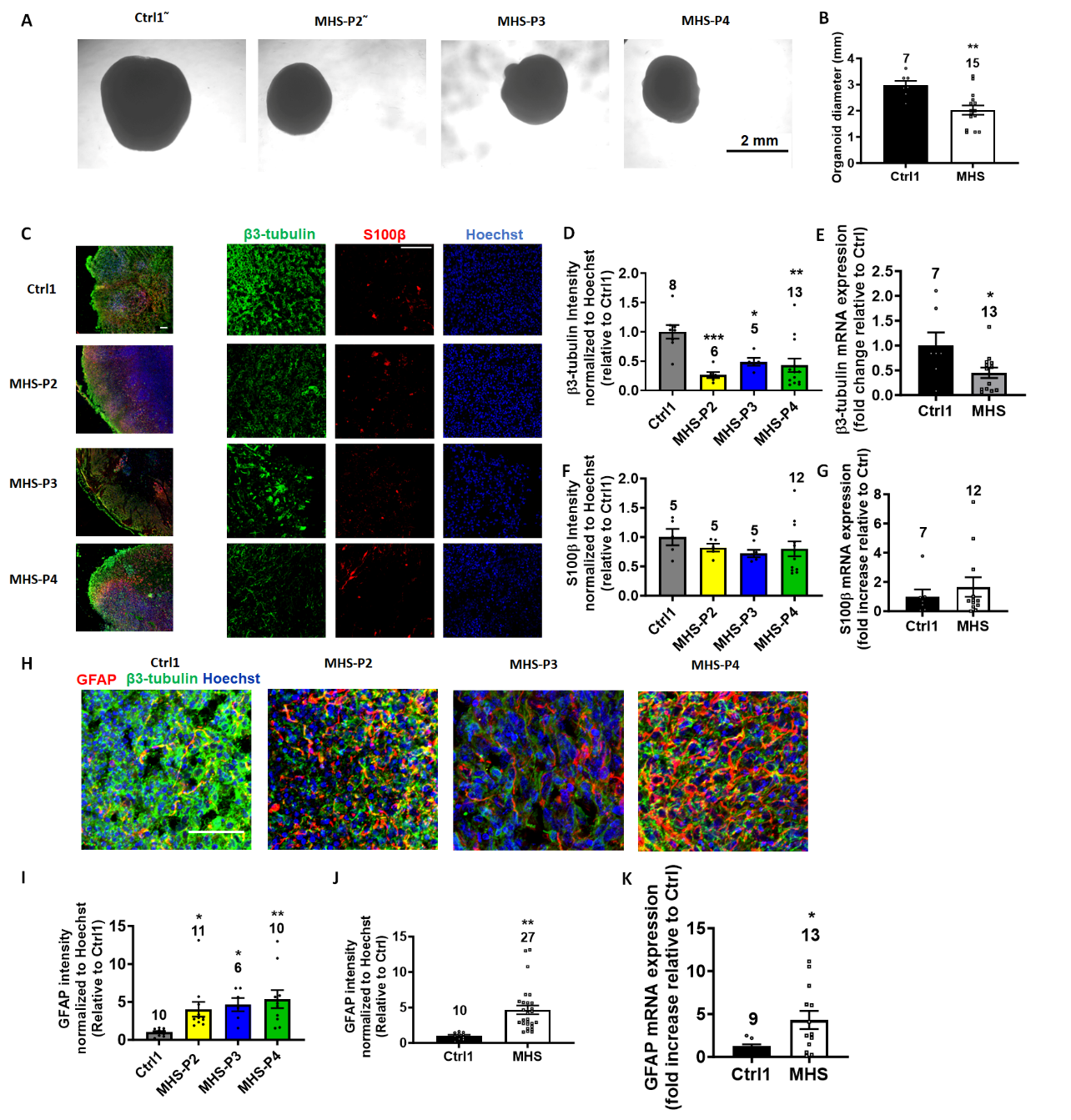
**

**Fig. S10 MHS hiPSC-derived cerebral organoids generate fewer neurons.**

**(A)** Representative phase-contrast images of 2-month-old Ctrl and MHS organoids. Scale bar, 2 mm.

(**B**) Quantification of the organoid diameter.

(**C**) Representative images of β3-tubulin, S100β, and Hoechst for Ctrl and MHS hiPSC-neurons. Left panel: Low power images of whole organoids. Right panel: Higher power images of individual fluorescence channels. Scale bar, 100 µm.

**(D)** Quantification of β3-tubulin fluorescence intensity for each MHS line normalized to Hoechst and relative to Ctrl.

**(E)** β3-tubulin mRNA expression by qRT-PCR for grouped MHS hiPSC-neurons vs. Ctrl.

**(F)** Quantification of S100β fluorescence intensity for each MHS line normalized to Hoechst and relative to Ctrl.

**(G)** S100β mRNA expression by qRT-PCR in Ctrl vs. MHS as a group.

(**H**) Representative images of GFAP, β3-tubulin, and Hoechst for Ctrl and MHS cerebral organoids. Scale bar, 50 µm.

(**I**) Quantification of GFAP fluorescence intensity for MHS cerebral organoids normalized to Hoechst and relative to Ctrl.

(**J**) Quantification of GFAP fluorescence intensity in Ctrl vs. MHS cerebral organoids as a group.

**(K**) GFAP mRNA expression by qRT-PCR in Ctrl vs. MHS organoids as a group. Data are mean + SEM. Number of imaged fields (n) from 3 separate cerebral organoids (C and E) or number of organoids analyzed by qRT-PCR (C, E and F) are listed above bars. **p* < 0.05; ***p* < 0.01; ****p* < 0.001 by ANOVA with Dunnett’s post hoc test for multiple comparisons or by unpaired Student’s t test for single comparisons.


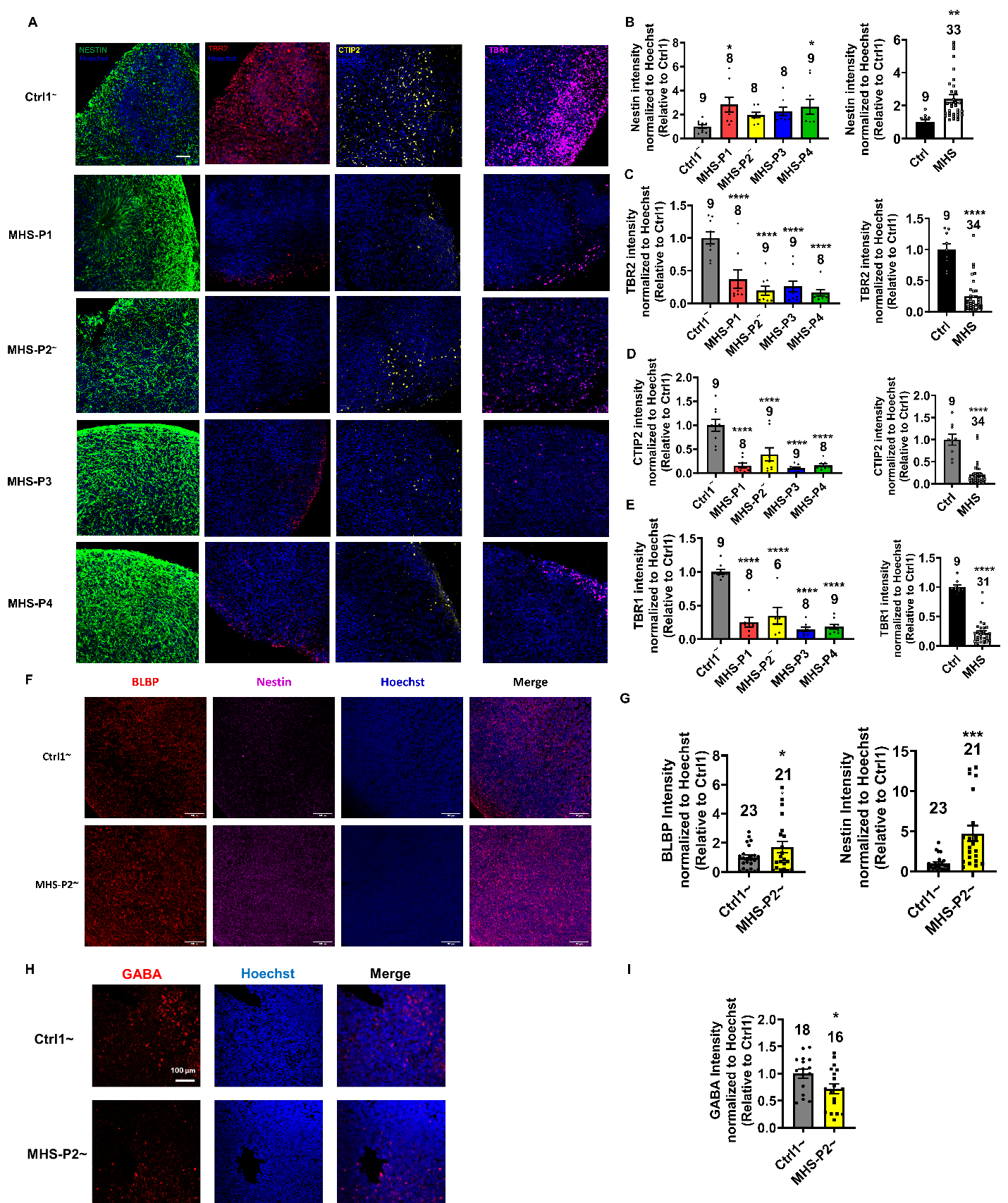


**Fig. S11 MHS hiPSC-derived cerebral organoids display disrupted layering and GABA expression.**

**(A)** Representative images from left to right of nestin, TBR2, CTIP2, TBR1, and Hoechst for Ctrl and MHS patients. Scale bar, 50 µm.

**(B)** Quantification of nestin fluorescence intensity for each MHS line relative to Ctrl. Fluorescence was normalized to Hoechst (labeling DNA in nucleus) to correct for cell number.

**(C)** TBR2 fluorescence intensity for each MHS line normalized to Hoechst and relative to Ctrl.

**(D)** CTIP2 fluorescence intensity for each MHS line normalized to Hoechst and relative to Ctrl.

**(E)** TBR1 fluorescence intensity for each MHS line normalized to Hoechst and relative to Ctrl.

**(F)** Representative images of BLBP and nestin staining for Ctrl and MHS cerebral organoids

**(G)** Quantification of BLBP (left) and nestin (right) fluorescence intensity in isogenic Ctrl vs. MHS cerebral organoids normalized to Hoechst. Data in bar graphs are mean ± SEM. Sample size is 3-4 sections from 4 separate cerebral organoids for each genotype. **p* < 0.05; ****p* < 0.001 by unpaired Student's t test.

**(H)** Representative images of GABA staining for Ctrl and MHS cerebral organoids

(**I**) Quantification of GABA fluorescence intensity in Ctrl vs. MHS cerebral organoids normalized to Hoechst. Data in bar graphs are mean ± SEM. Sample size is 3-4 sections from 3 separate cerebral organoids for each genotype. **p* < 0.05; *****p* < 0.0001 by ANOVA with Dunnett’s post hoc

**
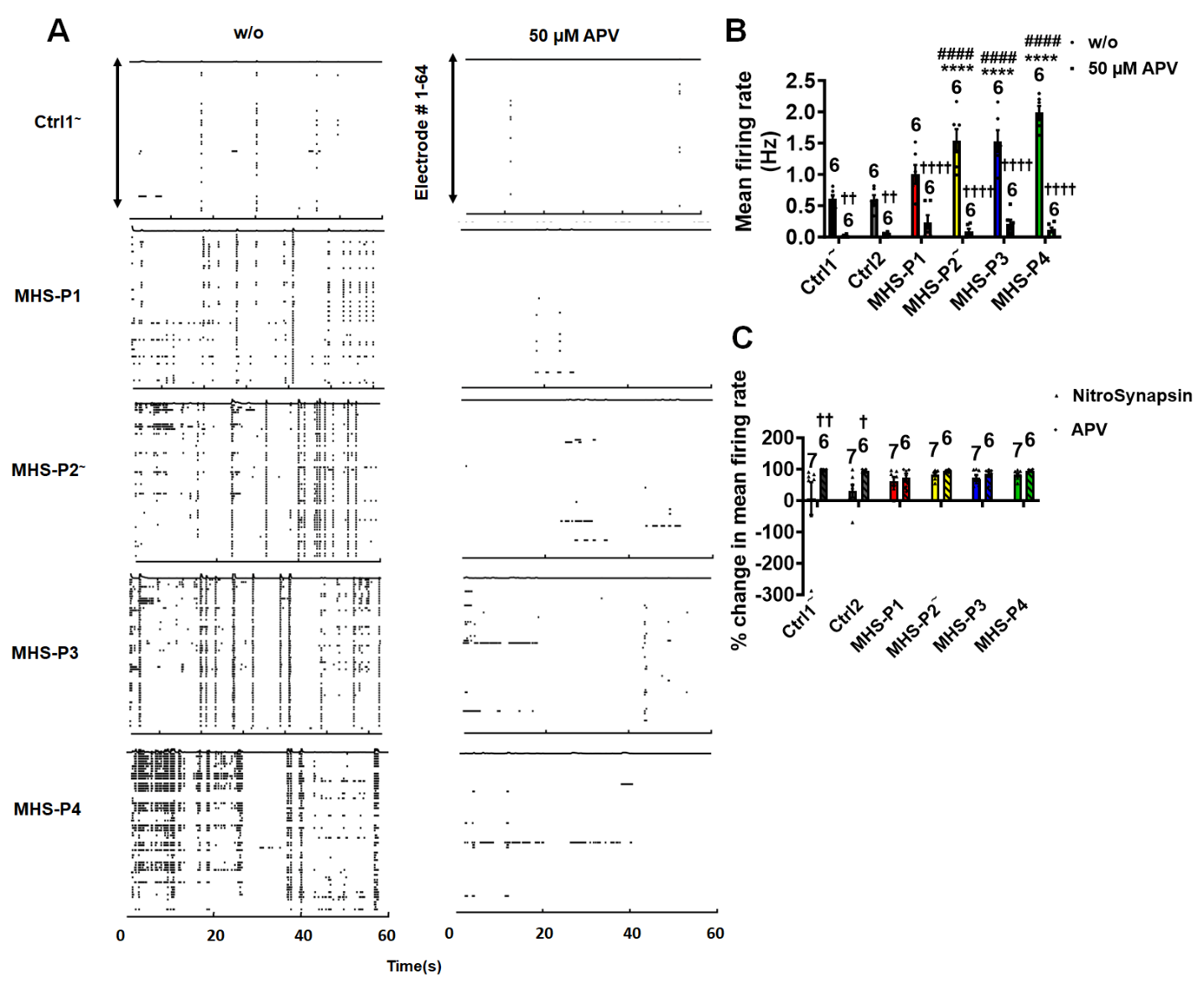
**

**Fig. S12 APV inhibits neuronal activity in MHS cerebral organoids.**

(**A**) Representative raster plots of MEA recordings in Ctrl and MHS cerebral organoids before and after treatment with 50 µM APV.

(**B**) Quantification of mean firing rate (Hz). Left (brighter) bar in each pair of bars represents data before APV treatment, while right (paler) bar of each pair represents data after APV treatment.

(**C**) Percentage inhibition in mean firing rate in the presence of NitroSynapsin (left/plain bar of each pair of bars) vs. APV (hashed/second bar of each pair). Data are mean ± SEM. Number of organoids listed above each bar for each genotype. *,^#,†^*p* < 0.05, ***^,###,†††^*p* < 0.001, ****^,####,††††^*p* < 0.0001 by ANOVA with Sidak’s post-hoc test for comparison to Ctrl1 (*) or to Ctrl2 (^#^). Comparisons within a genotype to compare with and without (w/o) APV treatment indicated by (^†^). In C, Mann-Whitney U test was used for single comparisons (see Star Methods).


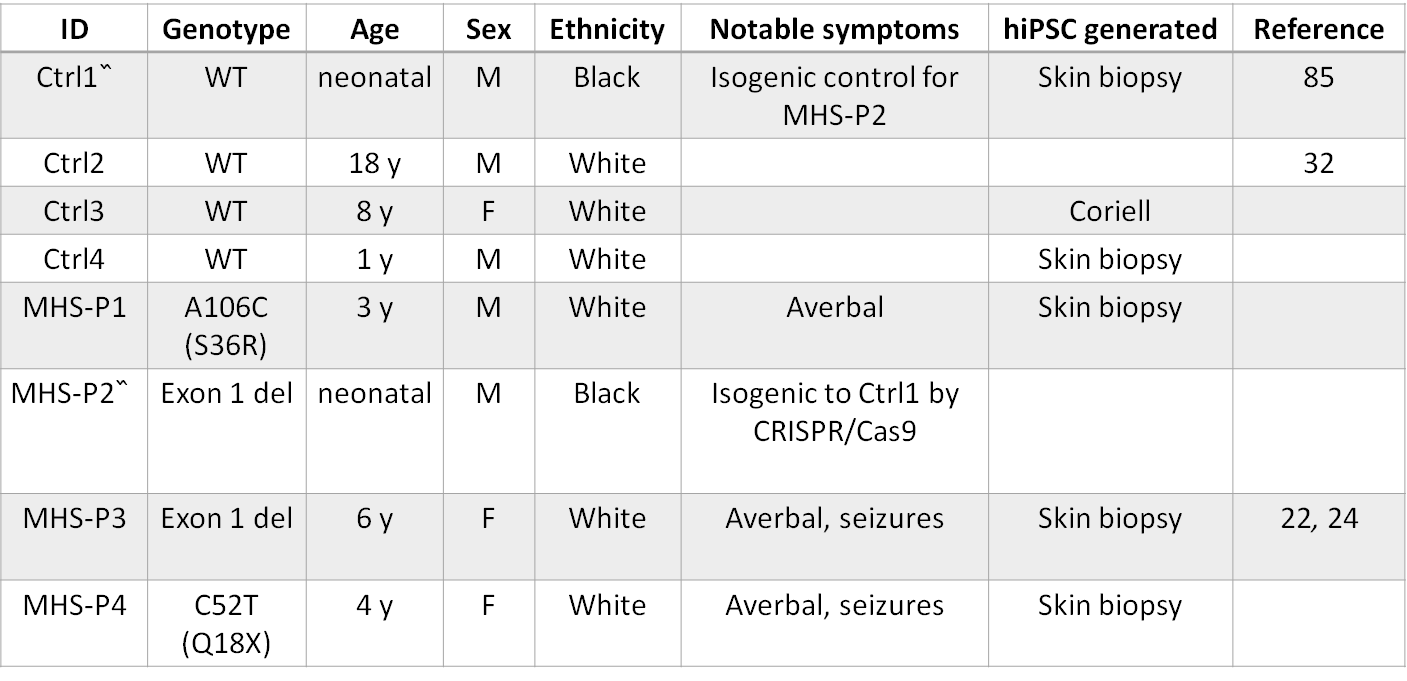


**Table S1 (above) Details of patients contributing fibroblasts for hiPSC generation.**

**Table S2**  **ChIP-seq targets of transcription factor MEF2** (see separate Excel file).

**
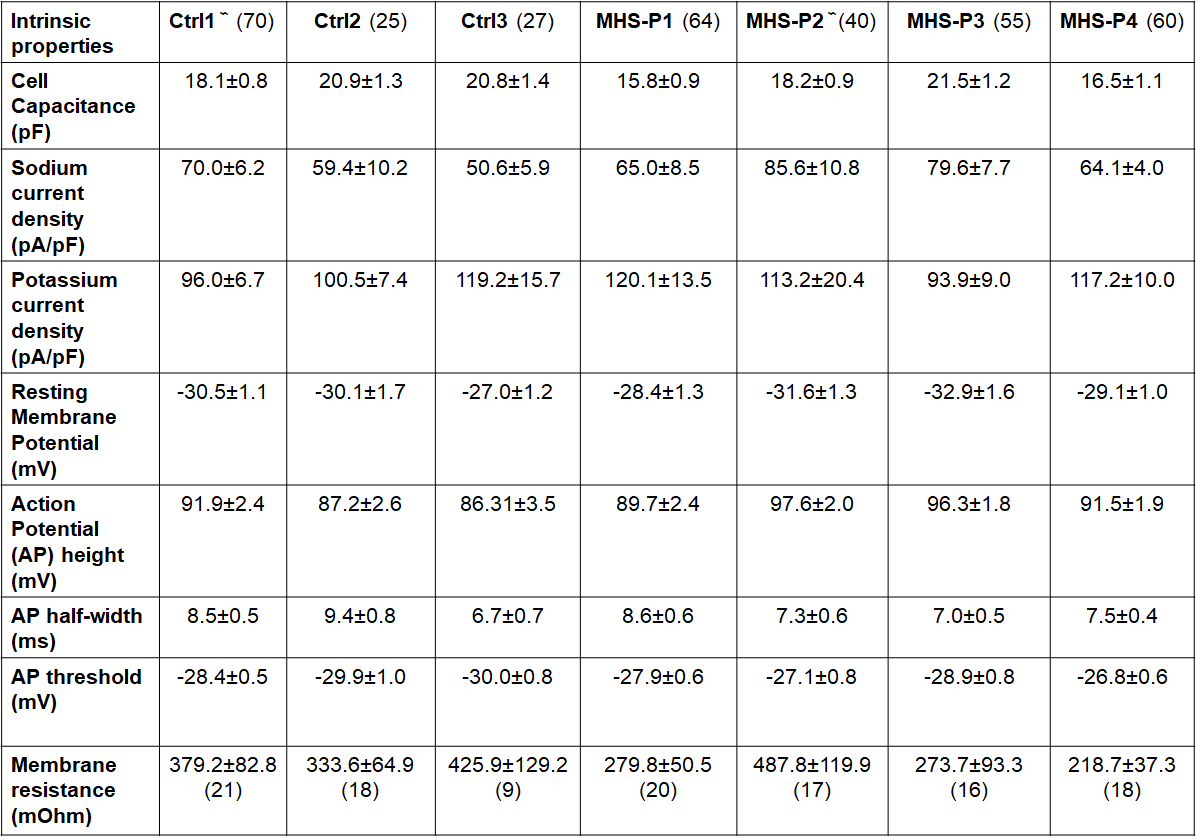
**

**Table S3 (above)** **Electrophysiological properties of control (Ctrl) and MHS patient hiPSC-derived cerebrocortical neurons in 2D culture.** Statistical comparisons were performed using ANOVA followed by a Sidak’s post-hoc test. The electrophysiological parameters listed in the table were not found to be significantly different among the various genotypes. The number of neurons analyzed per hiPSC line is listed in parentheses.

**Table S4**  **scRNA-seq data of cerebral organoids showing cell population by cluster analysis and relative expression of NRXN3** (see separate Excel file).

**Table S5**  **Top DEGs by cell type in MHS vs. isogenic WT cerebral organoids identified by scRNA-seq** (see separate Excel file).

**Video S1** **Ctrl hiPSC-derived cerebrocortical neuron and astrocyte 2D cultures.** Fluo-4 measurement of intracellular Ca^2+^ showing low basal endogenous signaling activity.

**Video S2 MHS hiPSC-derived cerebrocortical neuron and astrocyte 2D cultures.** Fluo-4 measurement of intracellular Ca^2+^ showing high synchronous burst-like basal endogenous signaling activity.

**Video S3** **Effect of NitroSynapsin on MHS hiPSC-derived cerebrocortical neuron and astrocyte 2D cultures.** Fluo-4 measurement of intracellular Ca^2+^ showing increased excitability, which was normalized after application of 10 µM NitroSynapsin.

**Additional supplementary information. Exact *p* values of the comparisons presented in the main and supplementary figures of the manuscript** (see separate .pdf file, Supplementary Information file 1).
