# Supplementary material for: Dysregulation of miRNA expression and excitation in MEF2C autism patient hiPSC-neurons and cerebral organoids
Source: Mol Psychiatry. 2024 Sep 30;30(4):1479–96. doi: 10.1038/s41380-024-02761-9 (PMC11919750; doi:10.1038/s41380-024-02761-9)
Supplement: Supplementary file 2 — Supplementary information file 1 [file 41380_2024_2761_MOESM2_ESM.pdf]

ADDITIONAL SUPPLEMENTARY INFORMATION:

Exact P values of the comparisons shown in the main figures.

<u>FIGURE 1</u>

Figure 1B:

ANOVA summary

P value	<0.0001
P value summary	****

Šídák's multiple comparisons test	Summary	Adjusted P Value
Ctrl1~ vs. MHS-P2~	*	0.0482
Ctrl1~ vs. MHS-P3	****	<0.0001
Ctrl1~ vs. MHS-P4	**	0.0024
Ctrl1~ vs. MHS-P1	**	0.0032
Ctrl2 vs. MHS-P2~	**	0.0038
Ctrl2 vs. MHS-P3	****	<0.0001
Ctrl2 vs. MHS-P4	***	0.0002
Ctrl2 vs. MHS-P1	***	0.0002

Figure 1C:

ANOVA summary

P value	<0.0001
P value summary	****

Šídák's multiple comparisons test	Summary	Adjusted P Value
Ctrl1~ vs. Ctrl2	ns	>0.9999
Ctrl1~ vs. Ctrl3	ns	>0.9999
Ctrl1~ vs. MHS-P2~	***	0.0002
Ctrl1~ vs. MHS-P3	****	<0.0001
Ctrl1~ vs. MHS-P1	**	0.0014
Ctrl1~ vs. MHS-P4	***	0.0009
Ctrl2 vs. MHS-P2~	***	0.0001
Ctrl2 vs. MHS-P3	****	<0.0001
Ctrl2 vs. MHS-P1	***	0.0008
Ctrl2 vs. MHS-P4	***	0.0006
Ctrl3 vs. MHS-P2~	**	0.0023
Ctrl3 vs. MHS-P3	***	0.0008
Ctrl3 vs. MHS-P1	*	0.0217
Ctrl3 vs. MHS-P4	**	0.0021
Ctrl2 vs. Ctrl3	ns	>0.9999

Figure 1C:

Unpaired t test

P value <0.0001

P value summary ****

Figure 1E:

ANOVA summary

P value 0.0102

P value summary *

**Dunnett's multiple comparisons test**	**Summary**	**Adjusted P Value**
~Ctrl1 vs. MHS-P1	ns	0.1272
~Ctrl1 vs. ~MHS-P2	*	0.012
~Ctrl1 vs. MHS-P3	**	0.0085
~Ctrl1 vs. MHS-P4	*	0.0324

Figure 1F:

Unpaired t test

P value 0.0199

P value summary *

Figure 1G:

ANOVA summary

P value <0.0001

P value summary ****

Šídák's multiple comparisons test	Summary	Adjusted P value

**Ctrl1˜ vs. Ctrl2**	**ns**	**0.1858**
**Ctrl1˜ vs. MHS-P1**	********	**<0.0001**
**Ctrl1˜ vs. MHS-P2˜**	********	**<0.0001**
**Ctrl1˜ vs. MHS-P3**	********	**<0.0001**
**Ctrl1˜ vs. MHS-P4**	********	**<0.0001**
**Ctrl2 vs. Ctrl3**	**ns**	**0.9429**
**Ctrl2 vs. MHS-P1**	********	**<0.0001**
**Ctrl2 vs. MHS-P2˜**	******	**0.0018**
**Ctrl2 vs. MHS-P3**	********	**<0.0001**
**Ctrl2 vs. MHS-P4**	********	**<0.0001**
**Ctrl3 vs. MHS-P1**	*******	**0.0009**
**Ctrl3 vs. MHS-P2˜**	*****	**0.0479**
**Ctrl3 vs. MHS-P3**	********	**<0.0001**
**Ctrl3 vs. MHS-P4**	********	**<0.0001**

FIGURE 2

Figure 2D:

ANOVA summary

P value	<0.0001
P value summary	****

Šídák's multiple comparisons test	Summary	Adjusted P Value
Ctrl (663a) vs. MHS (663a)	**	0.0019
Ctrl (663b) vs. MHS (663b)	**	0.0034
Ctrl (4273) vs. MHS (4273)	****	<0.0001

Figure 2E:

ANOVA summary

P value	0.0008
P value summary	***

Šídák's multiple comparisons test	Summary	Adjusted P Value
MHS-P1 control vs. MHS-P1 mimic 4273	*	0.0187
MHS-P2~ control vs. MHS-P2~ mimic 4273	**	0.0032
MHS-P4 control vs. MHS-P4 mimic 4273	*	0.0289

Figure 2F:

MHS-P1 4273 Mimic	Unpaired t test	
vs.	P value	0.0004
MHS-P1 control	P value summary	***

MHS-P2~ 4273 Mimic	Unpaired t test	
vs.	P value	0.0013
MHS-P2~ control	P value summary	**

MHS-P4 4273 Mimic	Unpaired t test	
vs.	P value	<0.0001
MHS-P4 control	P value summary	****

Figure 2G:

MHS-P1 4273 Mimic	Unpaired t test	
vs.	P value	0.0004
MHS-P1 control	P value summary	***

MHS-P2~ 4273 Mimic	Unpaired t test	
vs.	P value	0.0013
MHS-P2~ control	P value summary	**

MHS-P4 4273 Mimic	Unpaired t test	
vs.	P value	<0.0001
MHS-P4 control	P value summary	****

Figure 2L:

ANOVA summary

P value	0.0370
P value summary	*

Dunnett's multiple comparisons test	Summary	Adjusted P Value
Ctrl1~ vs. MHS-P2~	ns	0.2578
Ctrl1~ vs. MHS-P3	ns	0.7606
Ctrl1~ vs. MHS-P4	ns	0.9611
Ctrl1~ vs. MHS-P1	*	0.0144

FIGURE 3

Figure 3B:

ANOVA SUMMARY:

P value <0.0001

P value summary ****

Šídák's multiple comparisons test	Summary	Adjusted P Value
Ctrl1~ vs. Ctrl2	ns	0.9954
Ctrl1~ vs. Ctrl3	ns	>0.9999
Ctrl1~ vs. MHS-P1	ns	>0.9999
Ctrl1~ vs. MHS-P2~	*	0.0249
Ctrl1~ vs. MHS-P3	**	0.0051
Ctrl1~ vs. MHS-P4	*	0.0397
Ctrl2 vs. Ctrl3	ns	>0.9999
Ctrl2 vs. MHS-P1	ns	0.9999
Ctrl2 vs. MHS-P2~	***	0.0007
Ctrl2 vs. MHS-P3	***	0.0001
Ctrl2 vs. MHS-P4	**	0.0015
Ctrl3 vs. MHS-P1	ns	>0.9999
Ctrl3 vs. MHS-P2~	**	0.0092
Ctrl3 vs. MHS-P3	**	0.0021
Ctrl3 vs. MHS-P4	*	0.0141

Figure 3D:

ANOVA summary:

P value <0.0001

P value summary ****

Šídák's multiple comparisons test	Summary	Adjusted P Value
Ctrl1~ vs. Ctrl2	ns	>0.9999
Ctrl1~ vs. MHS-P1	ns	0.3304
Ctrl1~ vs. MHS-P2~	*	0.0426
Ctrl1~ vs. MHS-P3	****	<0.0001
Ctrl1~ vs. MHS-P4	**	0.0028
Ctrl2 vs. MHS-P1	ns	0.2575
Ctrl2 vs. MHS-P2~	*	0.0460
Ctrl2 vs. MHS-P3	***	0.0002
Ctrl2 vs. MHS-P4	**	0.0049

Figure 3E:

ANOVA summary:

P value <0.0001

P value summary ****

Šídák's multiple comparisons test	Summary	Adjusted P Value
Ctrl1~ vs. Ctrl2	ns	0.4974
Ctrl1~ vs. MHS-P1	****	<0.0001
Ctrl1~ vs. MHS-P2~	****	<0.0001
Ctrl1~ vs. MHS-P3	****	<0.0001
Ctrl1~ vs. MHS-P4	****	<0.0001
Ctrl2 vs. MHS-P1	***	0.0005
Ctrl2 vs. MHS-P2~	****	<0.0001
Ctrl2 vs. MHS-P3	***	0.0002
Ctrl2 vs. MHS-P4	***	0.0001

Figure 3F:

Kruskal-Wallis test

P value <0.0001

P value summary ****

Dunn's multiple comparisons test	Summary	Adjusted P Value
Ctrl1~ vs. Ctrl2	ns	>0.9999
Ctrl1~ vs. MHS-P1	*	0.0268
Ctrl1~ vs. MHS-P2~	**	0.0026
Ctrl1~ vs. MHS-P3	***	0.0001
Ctrl1~ vs. MHS-P4	***	0.0002
Ctrl2 vs. MHS-P1	*	0.0246
Ctrl2 vs. MHS-P2~	**	0.0024
Ctrl2 vs. MHS-P3	***	0.0001
Ctrl2 vs. MHS-P4	***	0.0002

Figure 3J:

ANOVA summary

P value 0.0001

P value summary ***

Dunnett's multiple comparisons test	Summary	Adjusted P Value
Ctrl1~ vs. MHS-P1	ns	0.3089
Ctrl1~ vs. MHS-P2~	**	0.0022
Ctrl1~ vs. MHS-P3	**	0.0014
Ctrl1~ vs. MHS-P4	****	<0.0001

Figure 3K:

ANOVA summary

P value <0.0001

P value summary ****

Dunnett's multiple comparisons test	Summary	Adjusted P Value
Ctrl1~ vs. MHS-P1	ns	0.6444
Ctrl1~ vs. MHS-P2~	**	0.0030
Ctrl1~ vs. MHS-P3	**	0.0013
Ctrl1~ vs. MHS-P4	***	0.0002

Figure 3L:

Kruskal-Wallis test

P value 0.9757

P value summary ns

Dunn's multiple comparisons test	Summary	Adjusted P Value
Ctrl1~ vs. MHS-P1	ns	>0.9999
Ctrl1~ vs. MHS-P2~	ns	>0.9999
Ctrl1~ vs. MHS-P3	ns	>0.9999
Ctrl1~ vs. MHS-P4	ns	>0.9999

FIGURE 4

Figure 4B:

ANOVA summary

P value 0.0186

P value summary *

Šídák's multiple comparisons test	Summary	Adjusted P Value
Ctrl1~ vs. Ctrl2	ns	0.5835
Ctrl1~ vs. Ctrl3	ns	>0.9999
Ctrl1~ vs. MHS-P1	ns	>0.9999
Ctrl1~ vs. MHS-P2~	ns	0.9948
Ctrl1~ vs. MHS-P3	*	0.0103
Ctrl1~ vs. MHS-P4	ns	0.9225
Ctrl2 vs. MHS-P1	ns	0.9741
Ctrl2 vs. MHS-P2~	ns	0.9986
Ctrl2 vs. MHS-P3	ns	0.7940
Ctrl2 vs. MHS-P4	ns	>0.9999
Ctrl3 vs. MHS-P1	ns	>0.9999
Ctrl3 vs. MHS-P2~	ns	>0.9999
Ctrl3 vs. MHS-P3	*	0.0380
Ctrl3 vs. MHS-P4	ns	0.9968

Figure 4C:

ANOVA summary

P value <0.0001

P value summary ****

Šídák's multiple comparisons test	Summary	Adjusted P Value
Ctrl1~ vs. Ctrl2	ns	0.9996
Ctrl1~ vs. Ctrl3	ns	>0.9999
Ctrl1~ vs. MHS-P1	ns	0.9921
Ctrl1~ vs. MHS-P2~	***	0.0001
Ctrl1~ vs. MHS-P3	**	0.0013
Ctrl1~ vs. MHS-P4	***	0.0008
Ctrl2 vs. Ctrl3	ns	>0.9999
Ctrl2 vs. MHS-P1	ns	>0.9999
Ctrl2 vs. MHS-P2~	**	0.0022
Ctrl2 vs. MHS-P3	*	0.0196
Ctrl2 vs. MHS-P4	*	0.0127
Ctrl3 vs. MHS-P1	ns	>0.9999
Ctrl3 vs. MHS-P2~	**	0.0012
Ctrl3 vs. MHS-P3	*	0.0114
Ctrl3 vs. MHS-P4	**	0.0073

Figure 4E:

ANOVA summary

P value 0.0678

P value summary ns

Šídák's multiple comparisons test	Summary	Adjusted P Value
Ctrl1~ vs. Ctrl2	ns	0.5476
Ctrl1~ vs. Ctrl3	ns	0.9992
Ctrl1~ vs. MHS-P1	ns	>0.9999
Ctrl1~ vs. MHS-P2~	ns	>0.9999
Ctrl1~ vs. MHS-P3	ns	0.9948
Ctrl1~ vs. MHS-P4	ns	>0.9999
Ctrl2 vs. Ctrl3	ns	0.0803
Ctrl2 vs. MHS-P1	ns	0.3460
Ctrl2 vs. MHS-P2~	ns	0.8249
Ctrl2 vs. MHS-P3	ns	0.9986
Ctrl2 vs. MHS-P4	ns	0.1139
Ctrl3 vs. MHS-P1	ns	>0.9999
Ctrl3 vs. MHS-P2~	ns	0.9745
Ctrl3 vs. MHS-P3	ns	0.5854
Ctrl3 vs. MHS-P4	ns	>0.9999

Figure 4F:

ANOVA summary

P value <0.0001

P value summary ****

Šídák's multiple comparisons test	Summary	Adjusted P Value
Ctrl1~ vs. Ctrl2	ns	>0.9999
Ctrl1~ vs. Ctrl3	ns	0.9783
Ctrl1~ vs. MHS-P1	****	<0.0001
Ctrl1~ vs. MHS-P2~	****	<0.0001
Ctrl1~ vs. MHS-P3	****	<0.0001
Ctrl1~ vs. MHS-P4	****	<0.0001
Ctrl2 vs. Ctrl3	ns	>0.9999
Ctrl2 vs. MHS-P1	***	0.0001
Ctrl2 vs. MHS-P2~	***	0.0008
Ctrl2 vs. MHS-P3	**	0.0010
Ctrl2 vs. MHS-P4	***	0.0001
Ctrl3 vs. MHS-P1	***	0.0008
Ctrl3 vs. MHS-P2~	**	0.0043
Ctrl3 vs. MHS-P3	**	0.0054
Ctrl3 vs. MHS-P4	***	0.0009

Figure 4H:

ANOVA summary

P value	0.0106
P value summary	*

Dunnett's multiple comparisons test	Summary	Adjusted P Value
Ctrl1~ vs. MHS-P2~	**	0.0064
Ctrl1~ vs. MHS-P4	*	0.0481
Ctrl1~ vs. MHS-P1	*	0.0143

Figure 4I:

ANOVA summary

P value	<0.0001
P value summary	****

Dunnett's multiple comparisons test	Summary	Adjusted P Value
Ctrl1~ vs. MHS-P2~	**	0.0042
Ctrl1~ vs. MHS-P4	****	<0.0001
Ctrl1~ vs. MHS-P1	****	<0.0001

Figure 4J:

ANOVA summary

P value	<0.0001
P value summary	****

Dunnett's multiple comparisons test	Summary	Adjusted P Value
Ctrl1~ vs. MHS-P2~	*	0.0101
Ctrl1~ vs. MHS-P4	**	0.0019
Ctrl1~ vs. MHS-P1	****	<0.0001

FIGURE 5

Figure 5B:

ANOVA summary

P value	<0.0001
P value summary	****

Šídák's multiple comparisons test	Summary	Adjusted P Value
Ctrl1~ vs. Ctrl2	ns	>0.9999
Ctrl1~ vs. MHS-P1	*	0.0437
Ctrl1~ vs. MHS-P2~	****	<0.0001
Ctrl1~ vs. MHS-P3	****	<0.0001
Ctrl1~ vs. MHS-P4	****	<0.0001
Ctrl2 vs. MHS-P1	ns	0.4498
Ctrl2 vs. MHS-P2~	****	<0.0001
Ctrl2 vs. MHS-P3	****	<0.0001
Ctrl2 vs. MHS-P4	****	<0.0001

Figure 5D:

Kruskal-Wallis test

P value	<0.0001
P value summary	****

Dunn's multiple comparisons test	Summary	Adjusted P Value
Ctrl1~ vs. Ctrl2	ns	>0.9999
Ctrl1~ vs. MHS-P1	*	0.0481
Ctrl1~ vs. MHS-P2~	**	0.0038
Ctrl1~ vs. MHS-P3	****	<0.0001
Ctrl1~ vs. MHS-P4	****	<0.0001
Ctrl2 vs. MHS-P1	***	0.0002
Ctrl2 vs. MHS-P2~	****	<0.0001
Ctrl2 vs. MHS-P3	****	<0.0001
Ctrl2 vs. MHS-P4	****	<0.0001

Figure 5E:

ANOVA summary

P value	<0.0001
P value summary	****

Šídák's multiple comparisons test	Summary	Adjusted P Value
Ctrl1~ vs. Ctrl2	ns	>0.9999
Ctrl1~ vs. MHS-P1	**	0.0015
Ctrl1~ vs. MHS-P2~	**	0.0015
Ctrl1~ vs. MHS-P3	*	0.0342
Ctrl1~ vs. MHS-P4	****	<0.0001
Ctrl2 vs. MHS-P1	**	0.0024
Ctrl2 vs. MHS-P2~	**	0.0024
Ctrl2 vs. MHS-P3	*	0.0484
Ctrl2 vs. MHS-P4	****	<0.0001

Figure 5G:

ANOVA summary

P value <0.0001

P value summary ****

Šídák's multiple comparisons test	Summary	Adjusted P Value
Ctrl1~ vs. Ctrl2	ns	>0.9999
Ctrl1~ vs. MHS-P1	**	0.0055
Ctrl1~ vs. MHS-P2~	***	0.0002
Ctrl1~ vs. MHS-P3	****	<0.0001
Ctrl1~ vs. MHS-P4	****	<0.0001
Ctrl2 vs. MHS-P1	*	0.0160
Ctrl2 vs. MHS-P2~	**	0.0010
Ctrl2 vs. MHS-P3	****	<0.0001
Ctrl2 vs. MHS-P4	****	<0.0001
Ctrl1~ vs. Ctrl1~+N	ns	>0.9999
Ctrl2 vs. Ctrl2+N	ns	>0.9999
MHS-P1 vs. MHS-P1+N	ns	0.0748
MHS-P2~ vs. MHS-P2~+N	**	0.0021
MHS-P3 vs. MHS-P3+N	**	0.0040
MHS-P4 vs. MHS-P4+N	***	0.0007

Figure 5H:

ANOVA summary

P value <0.0001

P value summary ****

Šídák's multiple comparisons test	Summary	Adjusted P Value
Ctrl1~ vs. Ctrl2	ns	>0.9999
Ctrl1~ vs. MHS-P1	**	0.0053
Ctrl1~ vs. MHS-P2~	***	0.0002
Ctrl1~ vs. MHS-P3	****	<0.0001
Ctrl1~ vs. MHS-P4	****	<0.0001
Ctrl2 vs. MHS-P1	***	0.0007
Ctrl2 vs. MHS-P2~	****	<0.0001
Ctrl2 vs. MHS-P3	****	<0.0001
Ctrl2 vs. MHS-P4	****	<0.0001
Ctrl1~ vs. Ctrl1~+N	ns	>0.9999
Ctrl2 vs. Ctrl2+N	ns	>0.9999
MHS-P1 vs. MHS-P1+N	*	0.0140
MHS-P2~ vs. MHS-P2~+N	*	0.0157
MHS-P3 vs. MHS-P3+N	***	0.0004
MHS-P4 vs. MHS-P4+N	***	0.0005

Figure 5I:

ANOVA summary

P value <0.0001

P value summary ****

Šídák's multiple comparisons test	Summary	Adjusted P Value
Ctrl1~ vs. Ctrl2	ns	>0.9999
Ctrl1~ vs. MHS-P1	*	0.0115
Ctrl1~ vs. MHS-P2	***	0.0003
Ctrl1~ vs. MHS-P3	****	<0.0001
Ctrl1~ vs. MHS-P4	****	<0.0001
Ctrl2 vs. MHS-P1	*	0.0489
Ctrl2 vs. MHS-P2	**	0.0020
Ctrl2 vs. MHS-P3	****	<0.0001
Ctrl2 vs. MHS-P4	****	<0.0001
Ctrl1~ vs. Ctrl1~+N	ns	>0.9999
Ctrl2 vs. Ctrl2+N	ns	>0.9999
MHS-P1 vs. MHS-P1+N	ns	0.2188
MHS-P2 vs. MHS-P2 +N	*	0.0268
MHS-P3 vs. MHS-P3+N	****	<0.0001
MHS-P4 vs. MHS-P4+N	***	0.0003

Figure 5J:

ANOVA summary

P value <0.0001

P value summary ****

Šídák's multiple comparisons test	Summary	Adjusted P Value
Ctrl1~ vs. MHS-P1	ns	0.3543
Ctrl1~ vs. MHS-P2~	**	0.0036
Ctrl1~ vs. MHS-P3	****	<0.0001
Ctrl1~ vs. MHS-P4	****	<0.0001
Ctrl1~ vs. Ctrl 2	ns	>0.9999
Ctrl 2 vs. MHS-P1	ns	0.5798
Ctrl 2 vs. MHS-P2~	*	0.0196
Ctrl 2 vs. MHS-P3	**	0.0013
Ctrl 2 vs. MHS-P4	***	0.0010
Ctrl1~ vs. Ctrl1~+N	ns	>0.9999
Ctrl 2 vs. Ctrl2+N	ns	>0.9999
MHS-P1 vs. MHS-P1+N	ns	0.5516
MHS-P2~ vs. MHS-P2~ +N	*	0.0279
MHS-P3 vs. MHS-P3+N	**	0.0012
MHS-P4 vs. MHS-P4+N	*	0.0481

FIGURE 7

Figure 7D:

ANOVA summary

P value	<0.0001
P value summary	****

Šídák's multiple comparisons test	Summary	Adjusted P Value
Ctrl1 vs. Ctrl2	ns	>0.9999
Ctrl1 vs. MHS-P1	ns	0.9445
Ctrl1 vs. MHS-P2	****	<0.0001
Ctrl1 vs. MHS-P3	**	0.0019
Ctrl1 vs. MHS-P4	***	0.0009
Ctrl2 vs. MHS-P1	ns	0.8669
Ctrl2 vs. MHS-P2	****	<0.0001
Ctrl2 vs. MHS-P3	**	0.0010
Ctrl2 vs. MHS-P4	***	0.0005
Ctrl1 vs. Ctrl1+N	ns	>0.9999
Ctrl2 vs. Ctrl2+N	ns	>0.9999
MHS-P1 vs. MHS-P1+N	ns	0.8984
MHS-P2 vs. MHS-P2+N	****	<0.0001
MHS-P3 vs. MHS-P3+N	**	0.0088
MHS-P4 vs. MHS-P4+N	**	0.0050

Figure 7F:

ANOVA summary

P value	<0.0001
P value summary	****

Šídák's multiple comparisons test	Summary	Adjusted P Value
Ctrl1 vs. Ctrl2	ns	0.9999
Ctrl1 vs. MHS-P1	ns	0.9801
Ctrl1 vs. MHS-P2	**	0.0035
Ctrl1 vs. MHS-P3	****	<0.0001
Ctrl1 vs. MHS-P4	****	<0.0001
Ctrl2 vs. MHS-P1	ns	0.5488
Ctrl2 vs. MHS-P2	***	0.0002
Ctrl2 vs. MHS-P3	****	<0.0001
Ctrl2 vs. MHS-P4	****	<0.0001
Ctrl1 vs. Ctrl+N	ns	>0.9999
Ctrl2 vs. Ctrl2+N	ns	>0.9999
MHS-P1 vs. MHS-P1+N	ns	0.7223
MHS-P2 vs. MHS-P2+N	**	0.0017
MHS-P3 vs. MHS-P3+N	****	<0.0001
MHS-P4 vs. MHS-P4+N	****	<0.0001

Figure 7H:

ANOVA summary

P value <0.0001

P value summary ****

Šídák's multiple comparisons test	Summary	Adjusted P Value
Ctrl1 vs. Ctrl2	ns	0.5747
Ctrl1 vs. MHS-P1	ns	0.9810
Ctrl1 vs. MHS-P2	***	0.0002
Ctrl1 vs. MHS-P3	****	<0.0001
Ctrl1 vs. MHS-P4	***	0.0006
Ctrl2 vs. MHS-P1	*	0.0356
Ctrl2 vs. MHS-P2	****	<0.0001
Ctrl2 vs. MHS-P3	****	<0.0001
Ctrl2 vs. MHS-P4	****	<0.0001
Ctrl1 vs. Ctrl1+N	ns	>0.9999
Ctrl2 vs. Ctrl2+N	ns	>0.9999
MHS-P1 vs. MHS-P1+N	ns	0.9981
MHS-P2 vs. MHS-P2+N	**	0.0057
MHS-P3 vs. MHS-P3+N	*	0.0206
MHS-P4 vs. MHS-P4+N	**	0.0079

Exact P values of the comparisons shown in the supplementary figures.

FIGURE S2

Figure S2C:

MHS	Unpaired t test	
vs.	P value	0.0396
Ctrl1	P value summary	*

Figure S2E:

MHS-P3	Unpaired t test	
vs.	P value	0.0001
Ctrl1	P value summary	***

Figure S2F:

ANOVA summary

**F**	**8.708**
**P value**	**<0.0001**
**P value summary**	**** **

Šídák's multiple comparisons test	Summary	Adjusted P Value
Ctrl1~ vs. Ctrl3	ns	0.9537
Ctrl1~ vs. MHS-P2~	****	<0.0001
Ctrl1~ vs. MHS-P3	*	0.0114
Ctrl1~ vs. MHS-P1	***	0.0002
Ctrl1~ vs. MHS-P4	ns	0.9109
Ctrl3 vs. MHS-P2~	**	0.0051
Ctrl3 vs. MHS-P3	ns	0.8507
Ctrl3 vs. MHS-P1	ns	0.3209
Ctrl3 vs. MHS-P4	ns	>0.9999

Figure S2G:

ANOVA summary

**P value**	**<0.0001**
**P value summary**	**** **

Šídák's multiple comparisons test	Summary	Adjusted P Value
Ctrl1~ vs. Ctrl3	ns	>0.9999
Ctrl1~ vs. MHS-P2~	***	0.0002
Ctrl1~ vs. MHS-P3	****	<0.0001
Ctrl1~ vs. MHS-P1	**	0.0013
Ctrl1~ vs. MHS-P4	***	0.0008
Ctrl3 vs. MHS-P2~	**	0.0021
Ctrl3 vs. MHS-P3	***	0.0008
Ctrl3 vs. MHS-P1	*	0.0173
Ctrl3 vs. MHS-P4	**	0.0019

<u>FIGURE S4</u>

Figure S4B:

ANOVA summary

P value **<0.0001**

P value summary ********

Šídák's multiple comparisons test	Summary	Adjusted P Value
ctrl 1 w/o vs. ctrl 1 TEA + 4AP	**	0.0022
ctrl 1 TEA + 4AP vs. ctrl1 w/o-TEA+4AP	*	0.0495
MHS-P2 w/o vs. MHS-P2 TEA+4AP	****	<0.0001
MHS-P2 TEA+4AP vs. MHS-P2 w/o-TEA+4AP	****	<0.0001

FIGURE S5

Figure S5E:

ANOVA summary

P value 0.0212

P value summary *

Dunnett's multiple comparisons test	Summary	Adjusted P Value
control vs. 663a inhibitor	*	0.0457
control vs. 4273 inhibitor	*	0.0342

Figure S5F:

ANOVA summary

P value 0.0580

P value summary ns

Dunnett's multiple comparisons test	Summary	Adjusted P Value
control vs. 663a inhibitor	*	0.0340
control vs. 4273 inhibitor	ns	0.2212

FIGURE S6

Figure S6B:

ANOVA summary

P value 0.0013

P value summary **

Dunnett's multiple comparisons test	Summary	Adjusted P Value
Ctrl1~ vs. MHS-P1	ns	0.9608
Ctrl1~ vs. MHS-P2~	ns	0.9356
Ctrl1~ vs. MHS-P3	**	0.0017
Ctrl1~ vs. MHS-P4	*	0.0332

Figure S6C:

ANOVA summary

P value <0.0001

P value summary ****

Dunnett's multiple comparisons test	Summary	Adjusted P Value
Ctrl1~ vs. MHS-P1	ns	0.0530
Ctrl1~ vs. MHS-P2~	****	<0.0001
Ctrl1~ vs. MHS-P3	***	0.0001
Ctrl1~ vs. MHS-P4	**	0.0033

Figure S6E:

ANOVA summary

P value 0.7269

P value summary ns

Dunnett's multiple comparisons test	Summary	Adjusted P Value
Ctrl1~ vs. MHS-P1	ns	0.5411
Ctrl1~ vs. MHS-P2~	ns	0.9980
Ctrl1~ vs. MHS-P3	ns	0.8387
Ctrl1~ vs. MHS-P4	ns	0.7967

Figure S6F:

ANOVA summary

P value <0.0001

P value summary ****

Dunnett's multiple comparisons test	Summary	Adjusted P Value
Ctrl1~ vs. MHS-P1	****	<0.0001
Ctrl1~ vs. MHS-P2~	****	<0.0001
Ctrl1~ vs. MHS-P3	****	<0.0001
Ctrl1~ vs. MHS-P4	****	<0.0001

FIGURE S7

Figure S7B:

ANOVA summary

P value <0.0001

P value summary ****

Dunnett's multiple comparisons test	Summary	Adjusted P Value
Ctrl1~ vs. Ctrl2	ns	0.9899
Ctrl1~ vs. MHS-P2~	*	0.0105
Ctrl1~ vs. MHS-P4	****	<0.0001
Ctrl1~ vs. MHS-P1	*	0.0396
Ctrl2 vs. MHS-P2~	**	0.0018
Ctrl2 vs. MHS-P4	****	<0.0001
Ctrl2 vs. MHS-P1	**	0.0070

FIGURE S8

Figure S8B:

ANOVA summary

P value	<0.0001
P value summary	****

Dunnett's multiple comparisons test	Summary	Adjusted P Value
Ctrl1~ w/o vs. MHS-P2~ w/o	*	0.0174
Ctrl1~ w/o vs. MHS-P3 w/o	*	0.0459
Ctrl1~ w/o vs. MHS-P4 w/o	****	<0.0001

Šídák's multiple comparisons test	Summary	Adjusted P Value
Ctrl1~ w/o vs. Ctrl1~ NitroSynapsin	ns	0.8172
MHS-P2~ w/o vs. MHS-P2~ NitroSynapsin	****	<0.0001
MHS-P3 w/o vs. MHS-P3 NitroSynapsin	****	<0.0001
MHS-P4 w/o vs. MHS-P4 NitroSynapsin	****	<0.0001

Figure S8D:

ANOVA summary

**P value**	**0.9119**
**P value summary**	**ns**

Šídák's multiple comparisons test	Summary	Adjusted P Value
Ctrl1+TTX vs. Ctrl1+TTX+NitroSyn	ns	0.9676
MHS-P2+TTX vs. MHS-P2+TTX+NitroSyn	ns	0.7654

MHS-P2+TTX	Unpaired t test	
vs.	P value	0.7295
Ctrl1+TTX	P value summary	ns

Figure S8D:

ANOVA summary

P value	0.0061
P value summary	**

Šídák's multiple comparisons test	Summary	Adjusted P Value
Ctrl1 vs. Ctrl1+APV	ns	0.8662
MHS-P2 vs. MHS-P2+APV	*	0.0168

MHS-P2	Unpaired t test	
vs.	P value	0.0222
Ctrl1	P value summary	*

FIGURE S10

Figure S10B:

MHS	Unpaired t test	
vs.	P value	0.0034
Ctrl1	P value summary	**

Figure S10D:

ANOVA summary

P value 0.0009

P value summary ***

Dunnett's multiple comparisons test	Summary	Adjusted P Value
Ctrl1 vs. MHS-P2	***	0.0007
Ctrl1 vs. MHS-P3	*	0.0277
Ctrl1 vs. MHS-P4	**	0.0016

Figure S10F:

ANOVA summary

P value 0.6086

P value summary ns

Dunnett's multiple comparisons test	Summary	Adjusted P Value
Ctrl1 vs. MHS-P2	ns	0.7325
Ctrl1 vs. MHS-P3	ns	0.4209
Ctrl1 vs. MHS-P4	ns	0.5475

Figure S10I:

ANOVA summary

P value 0.0086

P value summary **

Dunnett's multiple comparisons test	Summary	Adjusted P Value
Ctrl1 vs. MHS-P2	*	0.0481
Ctrl1 vs. MHS-P3	*	0.0433
Ctrl1 vs. MHS-P4	**	0.0039

Figure S10K:

MHS	Unpaired t test	
vs.	P value	0.0292
Ctrl1	P value summary	*

FIGURE S11

Figure S11B:

ANOVA summary

P value 0.0377

P value summary *

Dunnett's multiple comparisons test	Summary	Adjusted P Value
Ctrl1~ vs. MHS-P1	*	0.0181
Ctrl1~ vs. MHS-P2~	ns	0.3630
Ctrl1~ vs. MHS-P3	ns	0.1420
Ctrl1~ vs. MHS-P4	*	0.0316

Figure S11C:

ANOVA summary

P value <0.0001

P value summary ****

Dunnett's multiple comparisons test	Summary	Adjusted P Value
Ctrl1~ vs. MHS-P1	****	<0.0001
Ctrl1~ vs. MHS-P2~	****	<0.0001
Ctrl1~ vs. MHS-P3	****	<0.0001
Ctrl1~ vs. MHS-P4	****	<0.0001

Figure S11D:

ANOVA summary

P value <0.0001

P value summary ****

Dunnett's multiple comparisons test	Summary	Adjusted P Value
Ctrl1~ vs. MHS-P1	****	<0.0001
Ctrl1~ vs. MHS-P2~	****	<0.0001
Ctrl1~ vs. MHS-P3	****	<0.0001
Ctrl1~ vs. MHS-P4	****	<0.0001

Figure S11E:

ANOVA summary

P value <0.0001

P value summary ****

Dunnett's multiple comparisons test	Summary	Adjusted P Value
Ctrl1~ vs. MHS-P1	****	<0.0001
Ctrl1~ vs. MHS-P2~	****	<0.0001
Ctrl1~ vs. MHS-P3	****	<0.0001
Ctrl1~ vs. MHS-P4	****	<0.0001

Figure S11G (BLBP):

Unpaired t test

P value 0.0471

P value summary *

Figure S11G (Nestin):

Unpaired t test

P value 0.0003

P value summary ***

Figure S11I:

MHS-P2~	Unpaired t test	
vs.	P value	0.0336
Ctrl1~	P value summary	*

FIGURE S12

Figure S12B:

ANOVA summary

P value <0.0001

P value summary ****

Šídák's multiple comparisons test	Summary	Adjusted P Value
**Ctrl1** vs. **Ctrl2**	ns	>0.9999
**Ctrl1** vs. **MHS-P1**	ns	0.1441
**Ctrl1** vs. **MHS-P2**	****	<0.0001
**Ctrl1** vs. **MHS-P3**	****	<0.0001
**Ctrl1** vs. **MHS-P4**	****	<0.0001
**Ctrl2** vs. **MHS-P1**	ns	0.1161
**Ctrl2** vs. **MHS-P2**	****	<0.0001
**Ctrl2** vs. **MHS-P3**	****	<0.0001
**Ctrl2** vs. **MHS-P4**	****	<0.0001
**Ctrl1** vs. **Ctrl1+APV**	**	0.0023
**Ctrl2** vs. **Ctrl2+APV**	**	0.0050
**MHS-P1** vs. **MHS-P1+APV**	****	<0.0001
**MHS-P2** vs. **MHS-P2+APV**	****	<0.0001
**MHS-P3** vs. **MHS-P3+APV**	****	<0.0001
**MHS-P4** vs. **MHS-P4+APV**	****	<0.0001

Figure S12C:

Comparison	Test	Value
Ctrl1~+APV vs. Ctrl1~+NitroSynapsin	Mann Whitney test	
	P value	0.0023
	P value summary	**
Ctrl2+APV vs. Ctrl2+NitroSynapsin	Mann Whitney test	
	P value	0.0140
	P value summary	*
MHS-P1+APV vs. MHS-P1+NitroSynapsin	Mann Whitney test	
	P value	0.4452
	P value summary	ns
MHS-P2+APV vs. MHS-P2+NitroSynapsin	Mann Whitney test	
	P value	0.1807
	P value summary	ns
MHS-P3+APV vs. MHS-P3+NitroSynapsin	Mann Whitney test	
	P value	0.9452
	P value summary	ns
MHS-P4+APV vs. MHS-P4+NitroSynapsin	Mann Whitney test	
	P value	0.0734
	P value summary	ns
